# Integrated assessment of the impact of land use changes on groundwater recharge and groundwater level in the Drava floodplain, Hungary

**DOI:** 10.1038/s41598-022-21259-4

**Published:** 2023-03-28

**Authors:** Ali Salem, Yasir Abduljaleel, József Dezső, Dénes Lóczy

**Affiliations:** 1 Civil Engineering Department, Faculty of Engineering, Minia University, Minia, 61111 Egypt; 2 Doctoral School of Earth Sciences, University of Pécs, Ifjúság útja 6, 7624 Pecs, Hungary; 3 Department of Civil and Environmental Engineering, Washington State University, Richland, WA 99354 USA; 4 Faculty of Sciences, Institute of Geography and Earth Sciences, University of Pécs, Ifjúság útja 6, 7624 Pecs, Hungary

**Keywords:** Environmental sciences, Hydrology

## Abstract

Land-use/land-cover (LULC) change is considered a key human factor influencing groundwater recharge in floodplains. Without accurate estimations, the impact of LULC change on water balance components may be either significantly understated or exaggerated. This paper assesses the impacts of LULC changes from 1990 to 2018 on water balance components and groundwater levels of the Drava floodplain, Hungary, where human interference has led to a critical environmental situation. In this study, a spatially-distributed water balance model (WetSpass-M), and a groundwater flow model (MODFLOW-NWT) were integrated to assess the impacts of LULC changes. The moderate expansion of built-up areas increased surface runoff, while the afforestation of arable land and meadows and the overgrowth of bare mudflats with willow shrubs increased evapotranspiration. As a consequence, total annual groundwater recharge decreased by 5.3 × 10^7^ m^3^ in the floodplain with an average of 335 mm year^−1^ and 317 mm year^−1^ in 2012 and 2018, respectively. Moreover, an average groundwater level decline by 0.1 m is observed in the same period. Declined groundwater recharge, increased runoff, and evapotranspiration exerted a negative effect on water resources in the Drava basin. The approach tested in this paper allows temporal and spatial estimation of hydrological components under the changes of LULC, providing quantitative information for decision-makers and stakeholders to implement efficient and sustainable management of water resources in the Drava floodplain. The provided integrated model is also applicable to regionally.

## Introduction

The spatial distribution of groundwater reserves is highly influenced by human activities^1^, including land-use/land-cover (LULC) changes. The water balance components (i.e., actual evapotranspiration, groundwater recharge, and surface runoff) respond differently to LULC classes according to soil texture and climate conditions^2^. Therefore, their evaluation is a crucial and an urgent task for efficient water resources management and ecological restoration^3^.

The sustainable use of groundwater is based on groundwater recharge, which is one of the vital ecosystem services of floodplains and the basis of their efficient management^4^. Global climate change and human interventions substantially disturb the ecological regime and water balance of the floodplains^5^. River channelization and widespread agricultural utilization induced gradual desiccation of the Drava floodplain, reduced landscape diversity, and loss of wetlands^6^. In response to the gradual entrenchment of the Drava River channel and the loss of connectivity between the channel and the floodplain, droughts tend to occur with a frequency equal to that of floods^7,8^. For agricultural productivity, the dropping groundwater levels and the loss of root zone soil moisture are of severe consequences. The shortage of water and natural socio-economic changes have a substantial effect on the life quality of the local population^9^.

The evolution of groundwater recharge took place in different stages:

1. Initially, empirical and experimental methods like isotope tracers^10^, statistical methods such as the hydrological budget^11,12^ water-table fluctuation (WTF) method^13^.
2. In the last few decades, different numerical hydrological models^14,15^ have been developed to estimate the impacts of LULC on the water balance components^16–18^. Singh and Saraswat^17^ applied the soil and water assessment tool (SWAT) to assess the impacts of soil texture and LULC on water balance components. A simple daily soil–water balance (SWB) is developed to simulate recharge in the Mexico Basin^16^. The Système Hydrologique Européen has been further developed into MIKE SHE to be applied to ungauged basins^18^. The topographic hydrologic model (TOPMODEL) is found to work well for quantifying the surface runoff for mountain areas^19^.
3. Land-use models were also coupled with hydrological models, but their use is limited to flood prediction and surface runoff^20^.
4. An important further development was the water and energy transfer between soil, atmosphere, and plants, the water balance (WetSpass-M) model, which performs well regionally^21^ quantify the impacts of LULC change on the hydrological cycle^22,23^.

The paper’s novelty is an integrated framework model that couples water balance model (WetSpass-M) with the groundwater flow model (MODFLOW-NWT) for assessing the spatial distribution and the range of LULC change impacts on the hydrology of the floodplain. The main objectives of this study are:

1. to calibrate and validate the integrated framework model (WetSpass model through a comparison with Water Table Fluctuation method (WTF) and MODFLOW-NWT through observed groundwater levels);
2. to assess the impact of LULC change on groundwater recharge; and
3. to quantify the effect of LULC change on the total water budget and average groundwater level of the Drava floodplain.

## Site description

### Study area

**Figure 1:**
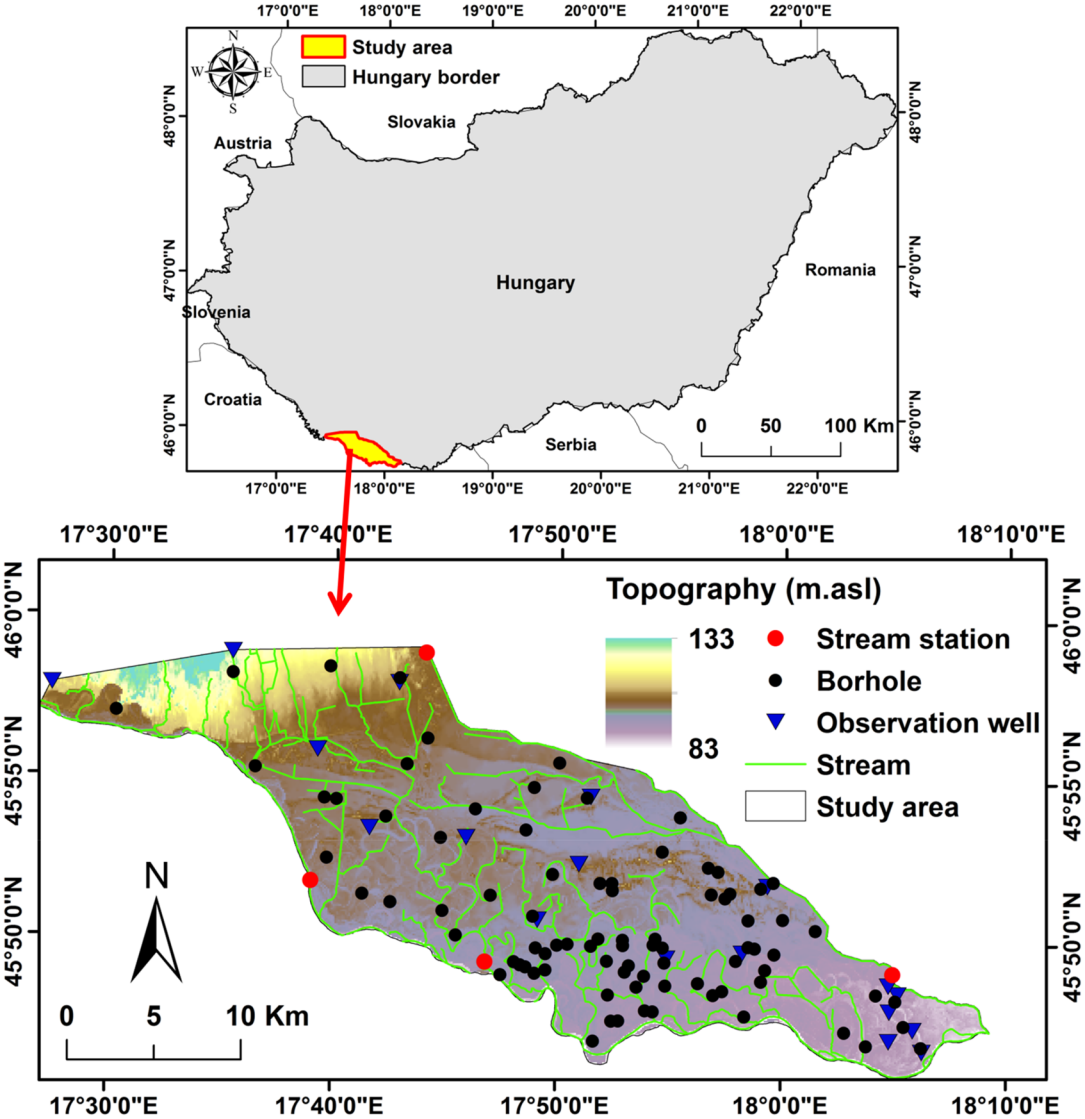
Location of the study area in Hungary, topography, streams, river gauges, boreholes and groundwater observation wells.

The investigated area covers the Drava floodplain region in the southwest part of Hungary [45.74°–46° N and 17.44°–18.15° E], with a total area of 538.46 km^2^ as shown in Fig. 1. It comprises 13 streams, 20 major channels, and 19 cut-off meanders with oxbow lakes^24^. Geologically, the Drava catchment is characterized by a variety of geological units from recent alluvial deposits in the lower Drava floodplain to old rocks in the center of the alpine basin. Their textural character is represented by coarse sand to heavy clay^9^. The Drava River is also located on the border between Croatia and Hungary in the axis of a 15-km-wide alluvial plain. The Drava is the largest river of the study area with a long-term average discharge of 595 m^3^ s^−1^ at Barcs station and an average water depth of 618 cm^25^.

## Materials and methods

### Input data

Table 1 presents the input parameters and their sources for the WetSpass-M model. All input data were prepared as raster maps with a resolution based on the digital elevation model (DEM) with cell size of 100 × 100 m totaling 547,249 raster cells (Fig. 1).

**Table 1:** Input data and sources for WetSpass-M model.

ID	Input parameter	Sources	Resolution
1	DEM and slope maps	STWMD and spatial analyst tools	100 × 100 m
2	Temperature map	STWMD and Kriging interpolation	100 × 100 m
3	Precipitation map	STWMD and Kriging interpolation	100 × 100 m
4	Wind speed map	STWMD and Kriging interpolation	100 × 100 m
5	PET map	STWMD and FAO-Penman–Monteith method	100 × 100 m
6	LULC maps	Corine data base (CLC 1990, 2000, 2006, 2012 and 2018)	100 × 100 m
7	Soil texture map	Thiessen polygon method for 89 geological bore holes	100 × 100 m
8	Groundwater depth	STWMD and Kriging interpolation of 18 observation wells	100 × 100 m
9	Land use parameters lookup table	WetSpass-M model	
10	Runoff coefficient lookup table	WetSpass-M model	
11	Soil parameter lookup table	WetSpass-M model	

**Figure 2:**
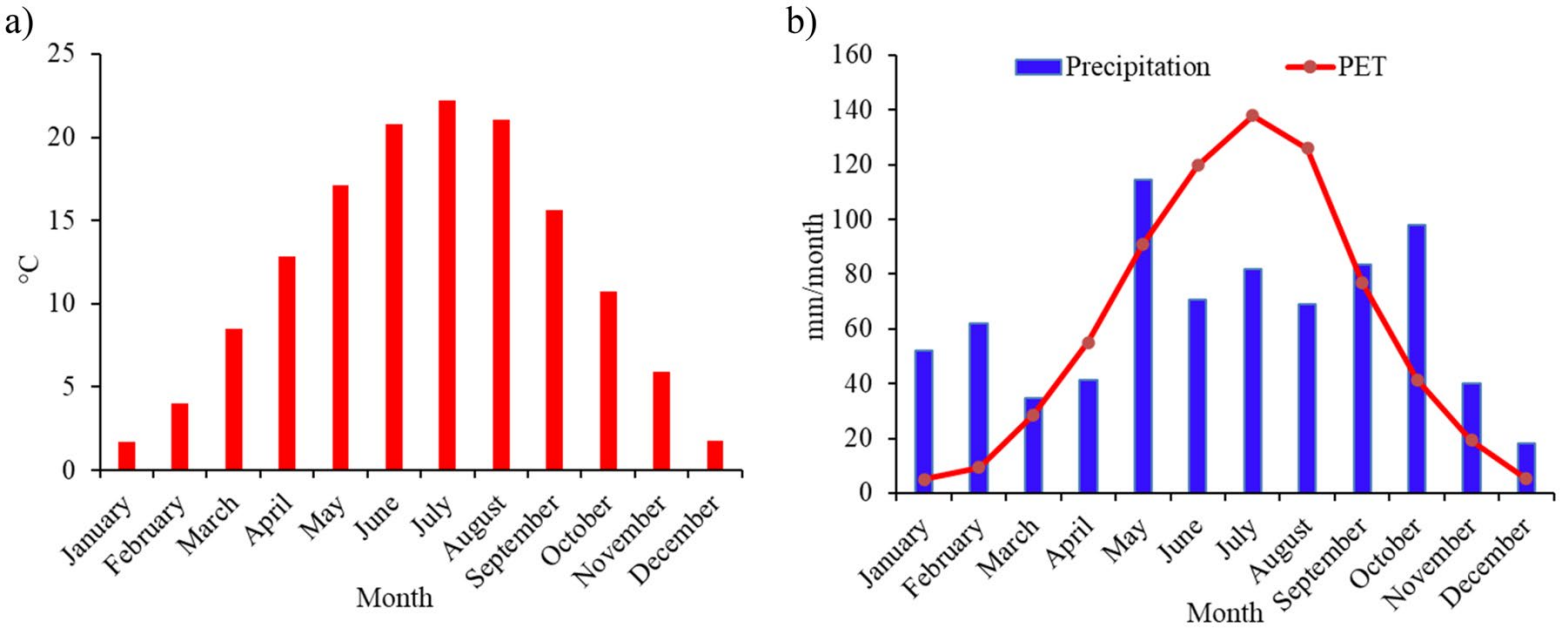
Long-term average monthly (1981–2018): (**a**) air temperature; (**b**) precipitation and potential evapotranspiration.

The floodplain climate is moderately warm and wet in the east and under a Mediterranean influence, while in the west it is under an Atlantic influence^26,27^. The average monthly air mean temperature for the period 2010 till 2018 is presented in Fig. 2a. The mean yearly global radiation is 4600–4700 MJ m^−2^. The precipitation changes considerably from month to month, as observed from Fig. 2b.

**Figure 3:**
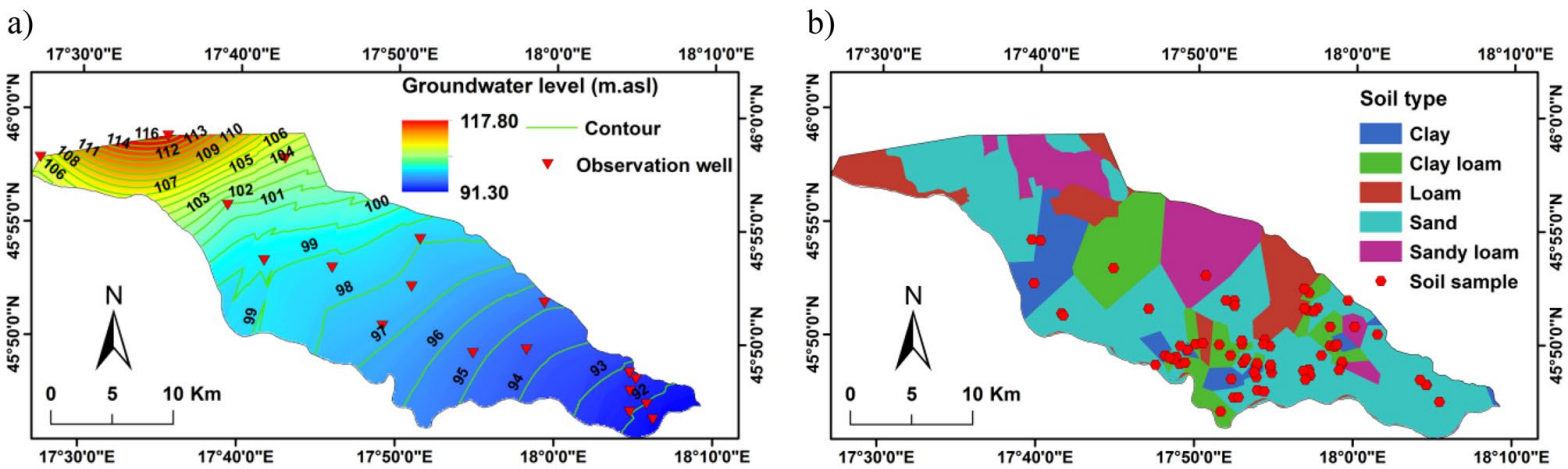
(**a**) Spatial distribution groundwater level; and (**b**) physical soil types of the study area.

PET was calculated using the FAO-Penman–Monteith method^28^ (Eq. (1)).

1 $$\text{PET }= \frac{0.408\Delta \left({R}_{n}-G\right)+\gamma \frac{900}{T+273}{u}_{2}({e}_{s}-{e}_{a})}{\Delta +\gamma (1+0.34{ u}_{2})},$$

where PET is the potential evapotranspiration (mm/day), ${R}_{n}$ is the net radiation (MJ m^−2^ day^−1^), G is the soil heat flux density (MJ m^−2^ day^−1^), ∆ is the slope of the saturation vapor pressure function (kPa °C^−1^), ${u}_{2}$ is the wind speed at 2 m height (m s^−1^), T is the daily average air temperature (°C), ${e}_{a} \,\text{is the actual vapor pressure }(\text{kPa}), {e}_{s}$ is the saturation vapor pressure (kPa), and γ is the psychometric constant (kPa °C^−1^). Average Monthly PET is shown in Fig. 2b.The spatial distribution of groundwater level was reconstructed using average daily groundwater level during the period from 2000 to 2018 from 18 observation wells (Fig. 3a).

The long-term average monthly leaf area index was downloaded from the Joint Research Center of the European Commission (JRCEC)^29^. Soils were sampled at 72 sites (Fig. 3b). The samples were dried in the oven at 60 °C till they reach constant weight. Malvern MasterSizer 3000HS, particle size analyzer, was used to perform the particle size analysis. The samples were classified into five textural classes of the WetSpass-M database. In the western part of the study area soil samples were obtained from the AGROTOPE database^30^. The spatial distribution of soil map was created using soil samples and AGROTOPE database (Fig. 3b) The dominant soil texture of the floodplain is sand (50% of the total area), followed by sandy loam (15%), loam (8%), clay loam (15%), and clay (12%).

The LULC maps for five time periods (1990, 2000, 2006, 2012, and 2018) were obtained from the CORINE database to evaluate the impacts of LULC change on groundwater recharge, groundwater head, and groundwater quantity. LULC maps are reclassed to 14 WetSpass-M classes, based on local expert knowledge.

The European CORINE Land Cover (CLC) mapping scheme is a project managed by the European Environment Agency. The CLC inventory was initiated in 1985, and updates have been developed in 2000, 2006, 2012, and 2018. The standard CLC nomenclature comprises of 44 land cover classes. These are classified in a three-level hierarchy. The first level consists of five main classes: (1) agricultural areas, (2) artificial surfaces, (3) wetlands, (4) forests and semi-natural areas, (5) water bodies^31^, the second level consists of 15 headings and the third, most detailed, level includes 44 thematic classes^32^. The satellite imagery, from which CLC vector layers were drawn, comprises of single date LANDSAT 5 MSS/TM (for CLC 1990) LANDSAT 7 Enhanced Thematic Mapper (ETM) ETM (for CLC2000), SPOT-4/5 and IRS P6 LISS III imagery (for CLC2006), IRS P6 LISS III and RapidEye (for CLC 2012), and lastly, Sentinel 2 and LANDSAT 8, for CLC (2018). CORINE provides a European scale map at 1:100,000, with a minimum mapping unit (MMU = 25 ha) for areal phenomena, 5 ha for change in land cover every 6 years^33^, minimum width of linear phenomena (MMW = 100 m) and thematic accuracy is higher than 85%. Those technical parameters are regarded as suitable for national or regional studies^34^. However, the CLC database has a particular limitation in spatial resolution, it is widely applied for environmental modelling, indicator development, and LULC change analysis in the European context^35^. CLC is considered one of the most important sources of the land cover database from a European perspective attributed to its applicability to large regions and the comparability of the landscape dynamics development of various landscape types^34^. Despite the above limitations, several studies have proved the reliability and applicability of using the CLC database for different purposes^35,36^.

### Modelling framework

**Figure 4:**
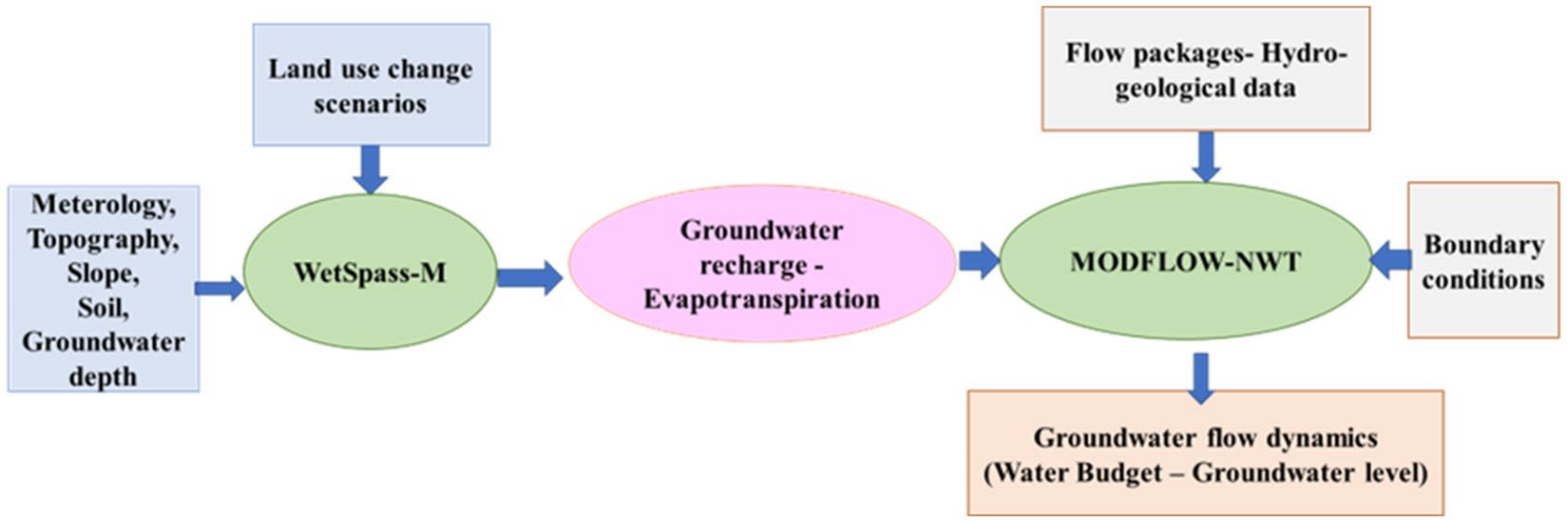
Adopted modelling framework.

WetSpass-M does not consider a two-way exchange between surface processes and groundwater, ignores run-on/runoff dynamics in recharge contribution. On the other hand, MODFLOW simulates flow processes occurring in the saturated zone defined by three-dimensional cells and the hydrogeological properties. To assess the effects of LULC changes on groundwater recharge and groundwater level, a link has been developed between LULC change and the groundwater system response. In this study, a modelling framework is formulated via coupling hydrological modelling (WetSpass-M) with the groundwater flow model (MODFLOW-NWT), applying ModelMuse as a graphical user interface, as shown in Fig. 4. In this framework, WetSpass simulates water balance components. Meanwhile, MODFLOW-NWT simulates three-dimensional groundwater flow and all associated sources and sinks (e.g. recharge, discharge to drains, interaction with lakes, and interaction with stream networks). Using this approach, the outputs of the WetSpass-M model (groundwater recharge and evapotranspiration) were used as inputs to MODFLOW-NWT. MODFLLOW-NWT computes groundwater hydraulic head and groundwater-surface water interactions. Such a coupled model was applied to evaluate the impacts of LULC changes on the hydrology (water balance components, water budget of the system, and groundwater level) of the Drava floodplain.

#### Hydrological modelling (WetSpass-M)

The spatially distributed water balance model WetSpass-M^21^ was applied to assess the impacts of changing LULC for the years (1990, 2000, 2006, 2012, and 2018) on groundwater recharge. The WetSpass-M model simulates monthly groundwater recharge, actual evapotranspiration, and surface runoff. The model’s values were used to evaluate the impacts of long-term LULC changes with the variability of soil texture on water balance components^37,38^. Moreover, it also works well at a regional scale^21^. The model includes the spatial distribution of land use, soil texture, slope, elevation, and meteorological parameters for each raster cell. Each raster cell is subdivided into a bare soil, vegetated, open water, and/or an impervious surface part. The water balance of each fraction was simulated using the following equation

2 $${\text{P }} = {\text{ R }} + {\text{ ET }} + {\text{ S}},$$

where P is the precipitation (LT^−1^), R is the groundwater recharge (LT^−1^), ET is the actual evapotranspiration (LT^−1^), and S is the surface runoff (LT^−1^).

The water balance of each raster cell was determined by summing up the independent water balances of each fraction per grid pixel. Total (ET) = f(vegetation coefficient and potential evapotranspiration)^39^. S = f(rainfall, interception, intensity, soil infiltration capacity, land use type, and soil texture, slope of the grid pixel). Groundwater recharge was determined as the residual parameter of the water balance. For mor details, see Abdollahi et al.^21^.

#### Groundwater flow model

MODFLOW-NWT^40^, with ModelMuse^41^ as a graphical user interface, was developed for a period of 9 years (2010–2018) on monthly time step to simulate the groundwater flow. MODFLOW-NWT model is particularly developed to solve problems caused by nonlinearities of drying and rewetting of the unconfined groundwater flow^40^. Groundwater system Inputs are groundwater recharge from lateral groundwater inflow from the northern boundary, precipitation, lake seepage, and river seepage while groundwater system outputs include groundwater seepage to lakes and rivers, groundwater evaporation, and lateral groundwater outflow from the southwestern boundary.

The topographic surface of the model is represented by the DEM. The model was vertically discretized in four layers based on the geological borehole information (Fig. 1). The model is discretized by a cell of a size of 30 m, and a total of 361 rows and 742 columns. The physical properties of the soils in the Drava floodplain are controlled by a swale-and-ridge topography^42^. The records of Ground Penetrating Radar (GPR), with auger holes, present high degree of heterogeneity in the hydraulic properties of the sediment^43^. The geological units of the investigated area range from coarse sand to clay. The hydraulic conductivity of each borehole was calculated using the falling head method. According to the geological boreholes data, the hydraulic conductivity of first layer characterized by four K-zones, ranging from 375 to 0.15 m day^−1^, while the second layer consisted of three zones, varying from 375 to 75 m day^−1^. The third and fourth layers were defined by two zones, ranging from 375 to 75 and 0.15 to 3 m day^−1^, respectively. Fortunately, the majority of boundaries are represented by a natural boundary. The Drava River and Fekete-víz Stream comprise the southern, eastern, northern, and part of the western boundary and these boundaries were parameterized by the River package. Also, a River package is used for all streams in the floodplain. The conductance of the riverbed is initially set to 5 m^2^ day^−1^ and, then, adjusted during the calibration process. The remaining part of the western boundary was assigned as a general head boundary (GHB) based on the records of two observation wells from the available 18 observation wells to simulate lateral inflow and outflow of groundwater in the floodplain. The conductance of GHB is assigned a value of 5 m^2^ day^−1^. The evapotranspiration and groundwater recharge boundaries were obtained from the results of the WetSpass-M model. More details of the model were described in Salem et al.^44^.

### Water fluctuation method

The water-table fluctuation method (WTF) is one of the most widely used techniques to estimate groundwater recharge. Here it is applied to validate and calibrate the WetSpass-M Model’s parameters. It needs data about changes in groundwater levels over time and specific yield. Recharge is then calculated by the following formula:

3 $$\text{R }= {\text{S}}_{\text{y}} \frac{\Delta \text{h}}{\Delta \text{t}},$$

where R is recharge (mm/month), S_y_ is specific yield, and ∆h is the change in water table high, and ∆t is time interval (month). Specific yield measurements are described in Appendix A. The measured values of specific yield for different lithologies are presented in Table S1. Table S2 presents the coordinates and annual change in groundwater level and specific yield for each of the observation wells.

## Results and discussion

### Calibration and validation of the WetSpass model

**Figure 5:**
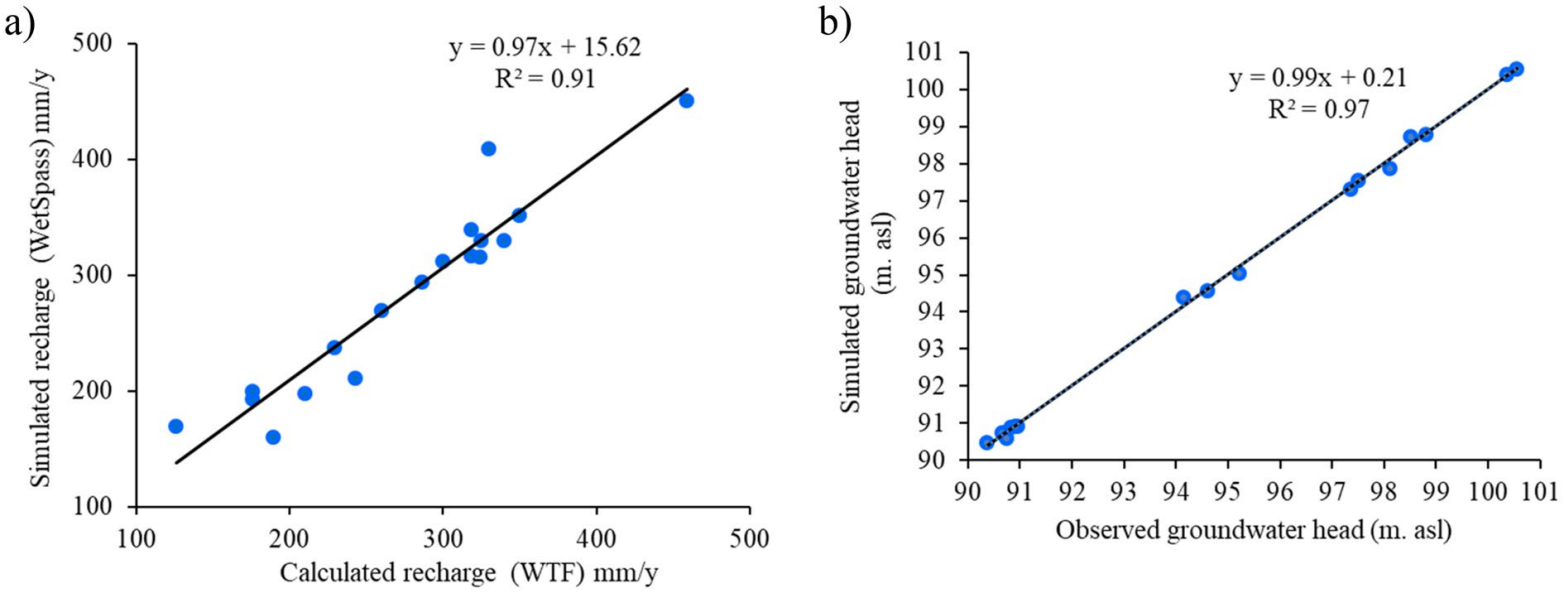
Scatter plot: (**a**) between the simulated recharge values (WetSpass-M) and calculated values (WTF) of groundwater recharge for 18 monitoring points; and (**b**) between simulated groundwater head and observed heads of 18 observation wells.

The model was calibrated using four parameters interception parameter (a), a calibration parameter (–) that decreases potential evapotranspiration based on soil moisture (LP), runoff delay factor (x), and soil moisture alfa coefficient (α). The four parameters were manually adjusted until obtaining the optimal match between simulated groundwater recharge from Wetspass-M and calculated groundwater recharge using Water Fluctuation method. The simulated groundwater recharge for the WetSpass-M model at the corresponding observed wells were extracted from the spatially distributed results in GIS. The calibrated values of a = 4.5, LP = 0.85, x = 0.5, and α = 1.5. Figure 5 shows that the simulated recharge by WetSpass-M matches the calculated recharge by WTF with R^2^ = 0.91, a mean error of 7 mm year^−1^, and an absolute mean error of 18 mm year^−1^. The simulated recharge by the WetSpass-M model was then considered conditionally validated.

### Calibration of the groundwater model

The groundwater model is developed by using the long-term average parameters, i.e. mean rainfall, mean PET and mean groundwater levels for the period from 1 January 2000 to 20 September 2018. The calibration process performed using average groundwater levels for 16 observation wells. The calibrated values of hydraulic conductivity result in six zones: 550, 170, 60, 5, 1.25, and 0.1 m day^−1^. The riverbed conductance for the main streams is 30 m^2^ day^−1^, and for secondary streams is 3 m^2^ day^−1^. The conductance of the GHB boundary retained the same values. Calibrated results of groundwater flow mode present a good agreement between observed and simulated groundwater heads with a mean error − 0.016 m and mean absolute error of 0.09 m as shown in (Fig. 5b).

### Water budget of the groundwater flow model

Table 2 presents the water balance of the calibrated model. Both groundwater recharge from precipitation and river are represented by 55% of the total inflow to the aquifer (Table 2). The total water budget of the floodplain proves the importance of groundwater recharge in maintaining groundwater levels. Of the total aquifer discharge 1,477,308 m^3^ day^−1^ (81%) is attributable to the river, while evapotranspiration represents 46,496 m3 day^−1^ (Table 2).

**Table 2:** The measured specific yield for different lithologies.

Lithology	Specific yield
Sand	0.27
Sandy loam	0.22
Loam	0.20
Clay loam	0.13
Clay	0.07

### Impacts of LULC changes on groundwater recharge

**Figure 6:**
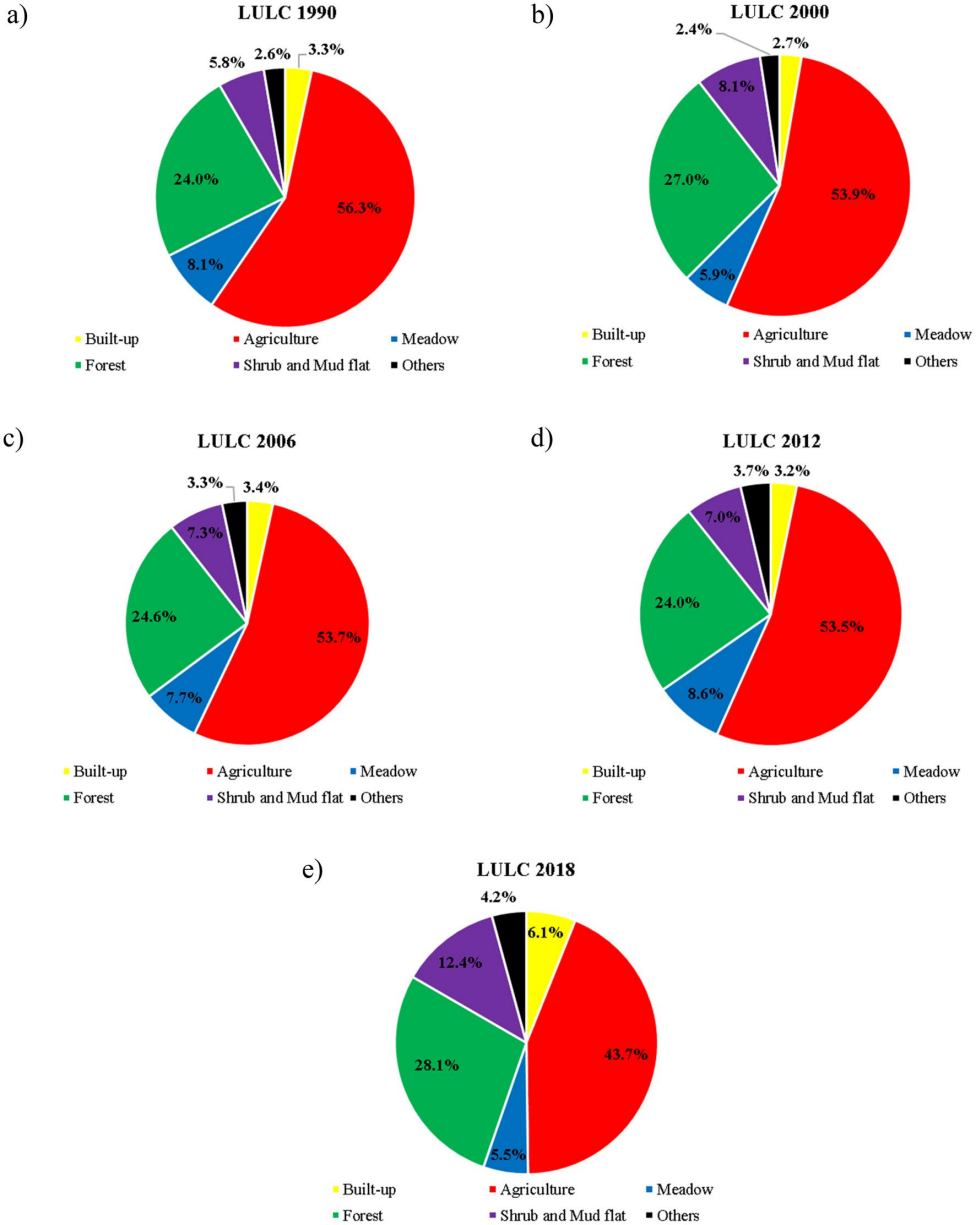
Area percentage of LULC classes in 1990, 2000, 2006, 2012, and 2018.

As depicted in Fig. S1, around half of the total area is under agriculture use, which is scattered over the whole floodplain. Agricultural areas are dominated by arable land (cereals and row crops). The extension of grassland (grazing land, meadow) is limited since animal husbandry has declined in the region. Forest areas are composed of coniferous forest, deciduous forest, and mixed forest with the dominance of deciduous forest. Coniferous forests are found in the southern part of the floodplain, while deciduous forests show a scattered pattern (Fig. S1). A comparison of LULC maps for the years 1990, 2000, 2006, 2012, and 2018 shows that the major changes observed in six LULC classes: Agriculture, forest, willow-poplar shrubs, urban, meadow, and mudflat during 1990–2018 (Fig. 6). The expansion of forests and mudflats occurred at the expense of arable land and meadow areas. Moreover, herbaceous vegetation is replaced gradually by willow and poplar shrubs.

**Figure 7:**
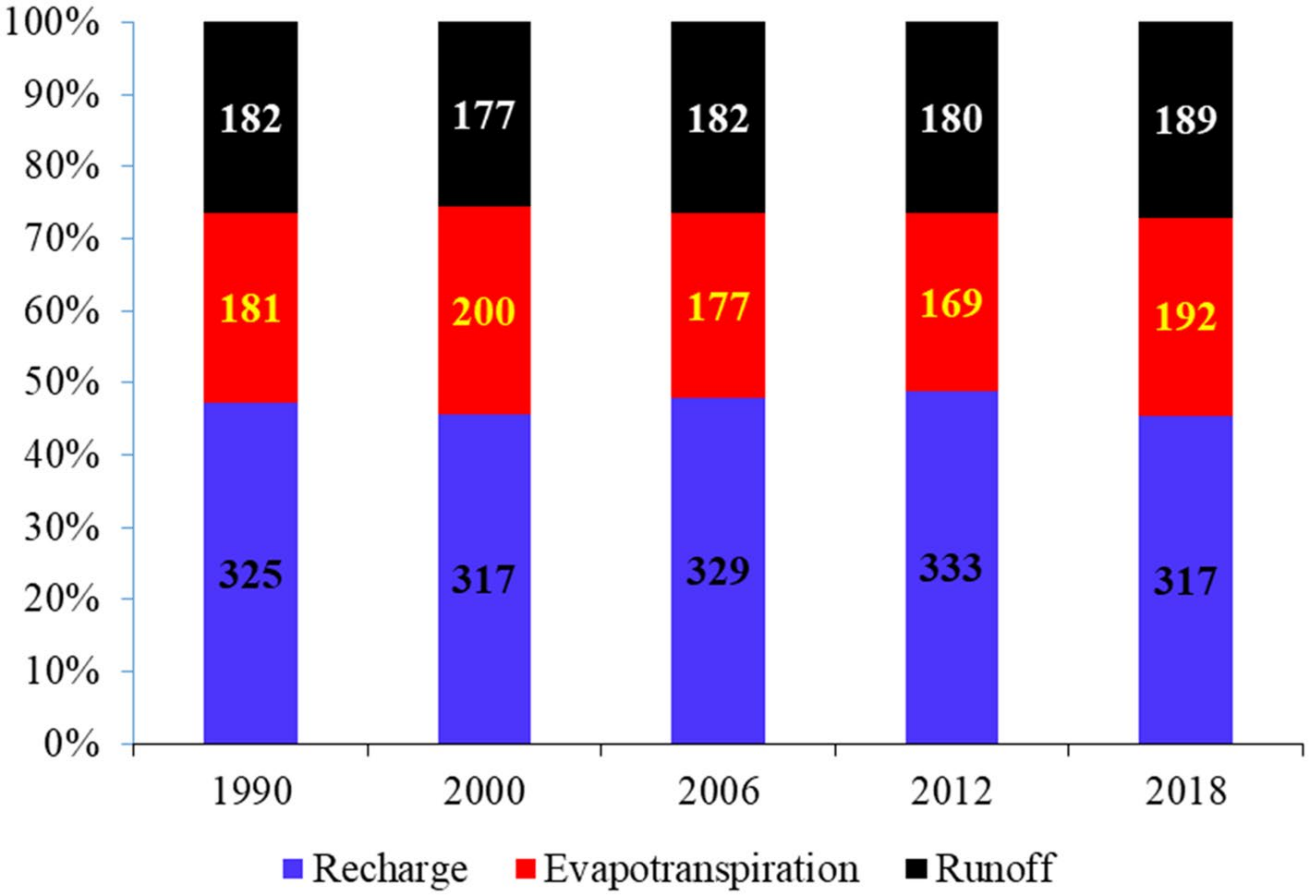
Average yearly water balance components for the Drava floodplain from 1990 to 2018 (values are in mm year^−1^).

Five different runs were launched, each corresponding to one LULC map from the years 1990, 2000, 2006, 2012, and 2018, respectively. To isolate, and yet capture, the effect of LULC changes, other input parameters such as meteorological data, topography (DEM and slope), distributed groundwater depth, and soil types were kept constant among the five runs. For the simulation meteorological variables for the period 2010–2018 was used as climate input, as well as the DEM, slope and soil of the study area for all the five runs. Each run simulated the long-term average water balance parameters in the 9 years from 2010 to 2018 (108 time steps). The simulated annual average water balance of the Drava floodplain for each LULC maps of the years 1990, 2000, 2006, 2012, and 2018 is presented in Fig. 7. The statistical analysis (minimum, maximum, average, standard deviation) for LULC of 1990, 2000, 2006, 2012, and 2018 is presented in Table S3. The spatial distribution of water balance components for the current scenario of 2018 is presented in Fig. S2.

As shown in Figs. 6 and 7, the decrease in surface runoff from 1990 and 2000 matches the decrease of built-up areas in this period. The comparison of changes in surface runoff and LULC maps indicates that the increase of average annual surface runoff by 9 mm year^−1^ can be attributed to built-up areas (sealed surface) expansion between 2012 and 2018. These areas are characterized by a partially or fully impervious surface and were considered as having a negative impact on the Drava floodplain. Changes in built-up areas were observed as the primary contributors for the change of surface runoff from 1990 to 2018.

The comparison between changes in actual evapotranspiration and groundwater recharge and changes of LULC indicated that changes of forest, willow—poplar shrubs, mudflat, meadow and arable land were the strongest contributor to the changes of recharge and evapotranspiration, suggesting the primary increase of evapotranspiration and decrease of recharge was attributed to the increase of forest, willow—poplar shrubs, mudflat and decrease of meadow areas and arable land from 1990 to 2000 and 2012 to 2018. The replacement of arable and meadow lands by forest, mudflat, with expanding willow shrub areas was identified as the strongest contributor to the major increase in evapotranspiration by 19 and 23 mm year^−1^ and a decrease in groundwater recharge by 8 and 16 mm year^−1^ between LCLU in 1990–2000 and 2012–2018, respectively (Fig. 7). The positive impact of forest shrubs on evapotranspiration is due to its relatively high transpiration demand compared to that of arable land and meadow areas. Conversely, from 2000 to 2012, the decrease of forest areas by 3% (27–24%), mudflat and shrubs areas from by 1.1% (8.1–7.0%), with expanding meadow areas by 2.7% (5.9–8.6%) (Fig. 6) were contributed to the major decrease in evapotranspiration by 31 mm year^−1^ and an increase in groundwater recharge by 16 mm year^−1^ (Fig. 7).

Unlike arable land, riparian willow shrubs have permanent access to shallow soil water and groundwater system^45,46^, increasing total leaf area of the canopy, evaporative, and transpiration losses^47,48^. This property of willow shrubs resulted in less water recharged. Indeed, mudflat areas are seasonally inundated by the river and have high groundwater levels, which enhanced transpiration and resulted in changes in the water balance of the floodplain by reducing groundwater recharge and increasing evapotranspiration. Moreover, urbanization leads to a decrease in groundwater recharge. Because of low groundwater and high drought hazard ecosystem services are provided at a lower level, and agricultural productivity is reduced because of the water deficit. Thus, the agricultural productivity and ecosystem services in the Drava floodplain reached a critical situation and, hence, human activities have a negative net effect on groundwater recharge in the floodplain. Thus, understanding the spatial distribution of groundwater recharge changes is crucial for water resources management in the Drava floodplain.

Minimum, maximum, average, and standard deviation percentages of change in groundwater recharge of the simulated LULC scenarios (2000, 2006, 2012, and 2018) with respect to the base year 1990 are presented in Table 3. The difference in groundwater recharge which occurred between 1990 and 2018 ranged from − 100 to 289.7%, with an average decrease of − 2.6%. The spatial distribution of the change in simulated groundwater recharge for the LULC scenarios is shown in Fig. S3.

**Table 3:** Minimum, maximum, average, and standard deviation values of the change in groundwater recharge in % of the simulated LULC scenarios (2000, 2006, 2012 and 2018) with respect to the base LULC in 1990.

	Change in groundwater recharge			
	2000–1990	2006–1990	2012–1990	2018–1990
	%			
Minimum	− 100	− 100	− 100	− 100
Maximum	256.8	273.6	273.6	289.7
Average	− 2.5	1.3	2.4	− 2.6
St.dev	12.2	14.5	16.3	30.9

**Figure 8:**
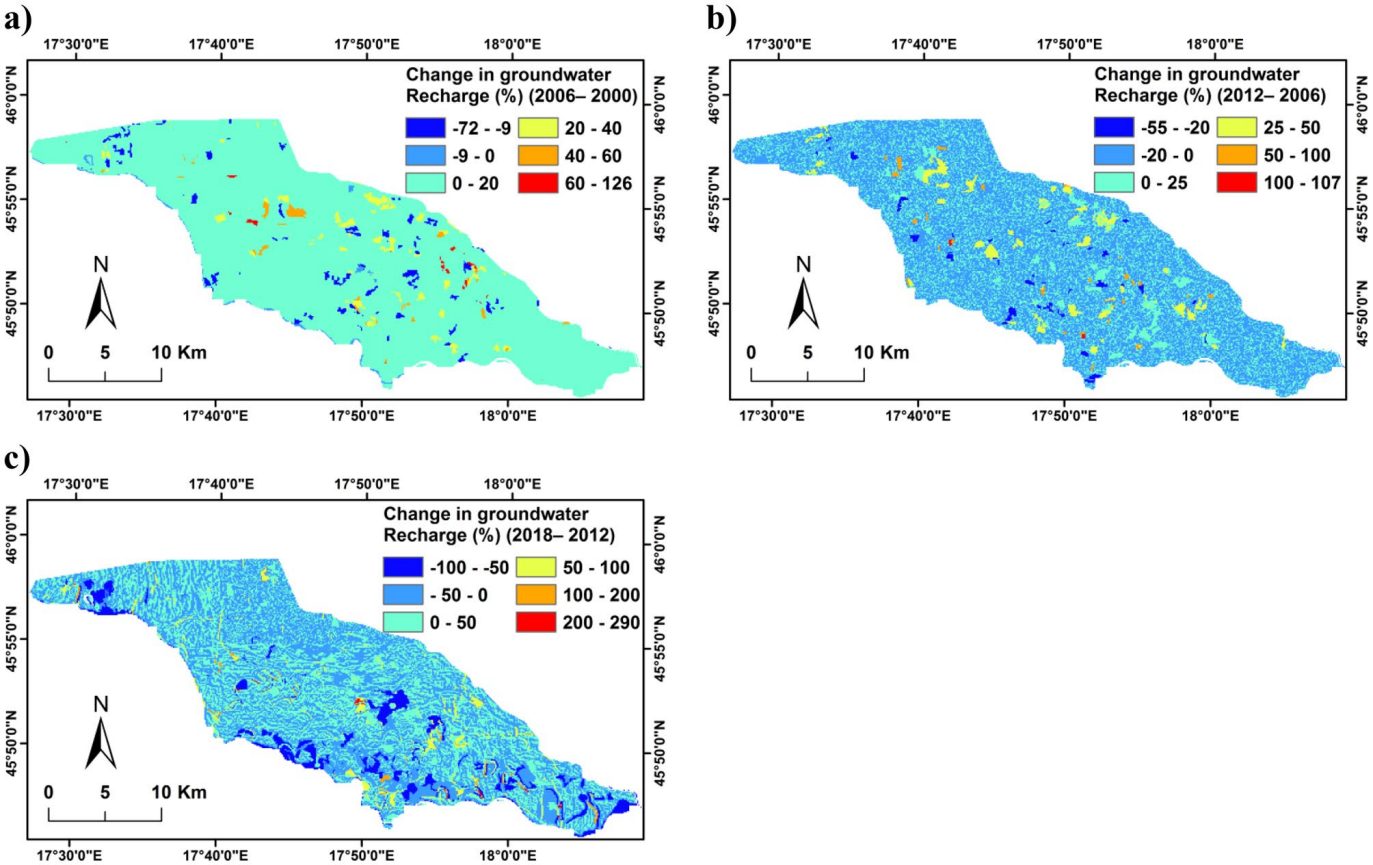
Change in groundwater recharge: (**a**) 2006–2000; (**b**) 2012–2006; (**c**) 2018–2012.

Moreover, the change in groundwater recharge was investigated between 2000 and 2018 with an interval of 6 years. In a time period from 2000 to 2006, the average change in groundwater recharge was 4% (Fig. 8a). This variation of groundwater recharge associate with changes in arable land, mudflat, forest and meadow. A small variation in groundwater recharge was observed during 2006–2012 with an average ratio of 1% due to small changes in LULC for this period (Fig. 8b). As depicted in Fig. 8c, the average changes in groundwater recharge from 2000 to 2006 was − 5%. This means that the total groundwater recharge of LULC in 2018 was decreased by 5.3 × 10^7^ m^3^ with respect to LULC in 2012. This reduction in groundwater recharge is associated with the expansion of built-up areas, forest, mudflat, and growing of willow shrubs with shrinkage of agriculture areas. The results clearly indicate that LULC changes affect the overall water budget of the floodplain. The applied approach used in this study allows temporal and spatial estimation of hydrological components under the changes of LULC, providing quantitative information for decision-makers and stakeholders to implement an efficient and sustainable management of water resources in Drava floodplain.

### Water balance components under different land uses and soil textures

**Figure 9:**
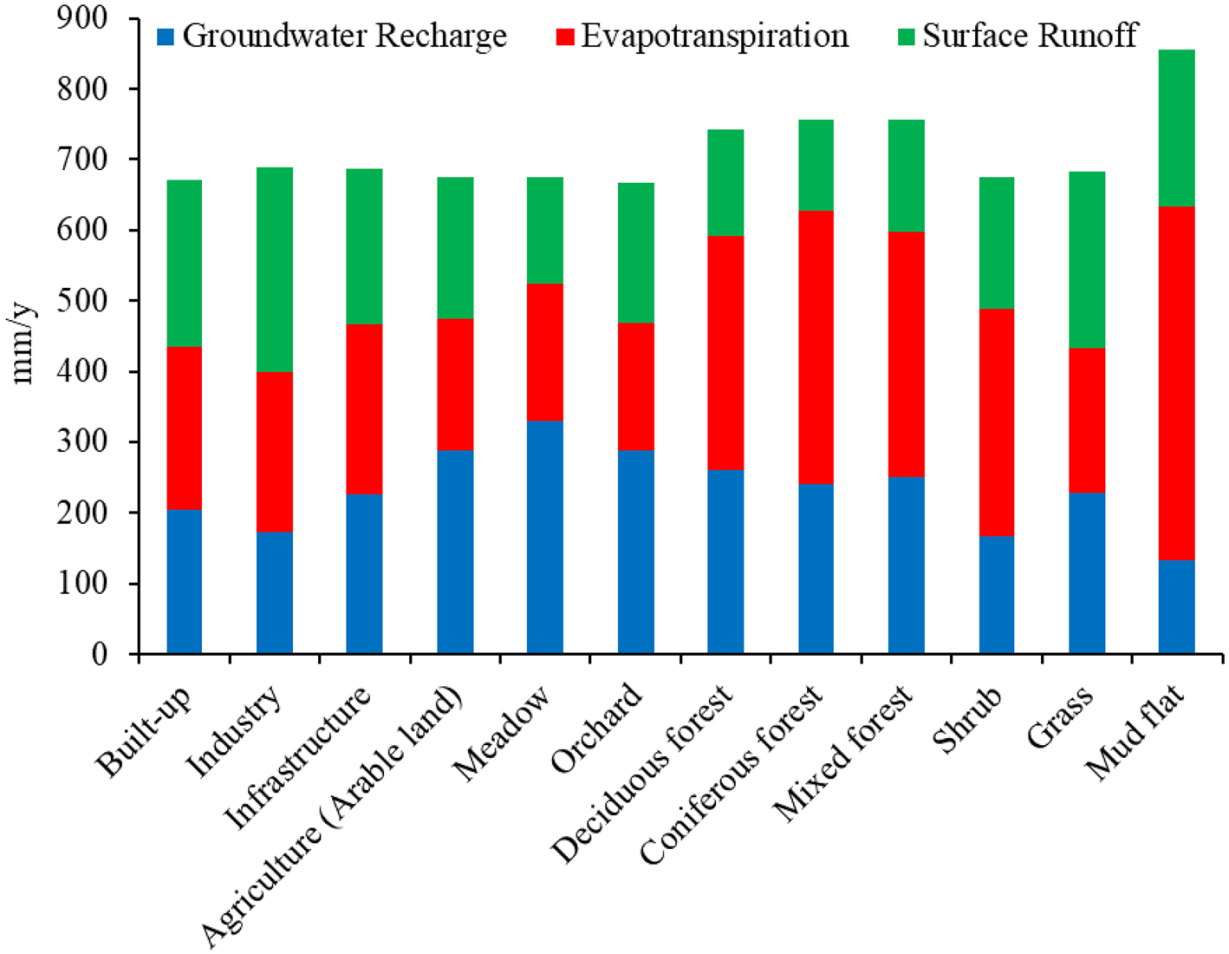
Average annual groundwater recharge, surface runoff and evapotranspiration as a function of LULC in 2018.

LULC changes from 1990 to 2018 are considered the major factor contributing to the spatial variation of actual evapotranspiration, groundwater recharge, and surface runoff in the Drava floodplain. To quantify these impacts, we assessed water balance components under different LULC classes. Figure 9 shows the average annual groundwater recharge, runoff, and actual evapotranspiration, as a function of LULC classes under LULC of 2018 in the Drava floodplain. Groundwater recharge, actual evapotranspiration, and surface runoff are strongly dependent on LULC. Mudflats (deeper lying areas which are seasonally inundated by the river and have high groundwater levels) and forests show high average actual evapotranspiration by 499 and 322 mm year^−1^, respectively, while they have average groundwater recharges of 133 and 251 mm year^−1^, respectively. Groundwater recharge is assigned to a value of zero for open water surfaces (i.e., rivers and lakes) in the WetSpass-M model since the open water surfaces are assumed to be groundwater discharge locations^49^. Similar to a study of the southern Moravian floodplain forest by Čermák and Prax^50^, forest areas are characterized by high actual evapotranspiration with an average of 322 mm year^−1^. The coniferous forest has the lowest surface runoff with an average of 130 mm year^−1^. Built-up areas show a low groundwater recharge and actual evapotranspiration with an average of 204 and 231 mm year^−1^, respectively, as these areas are characterized by a partially or fully impervious surface.

**Figure 10:**
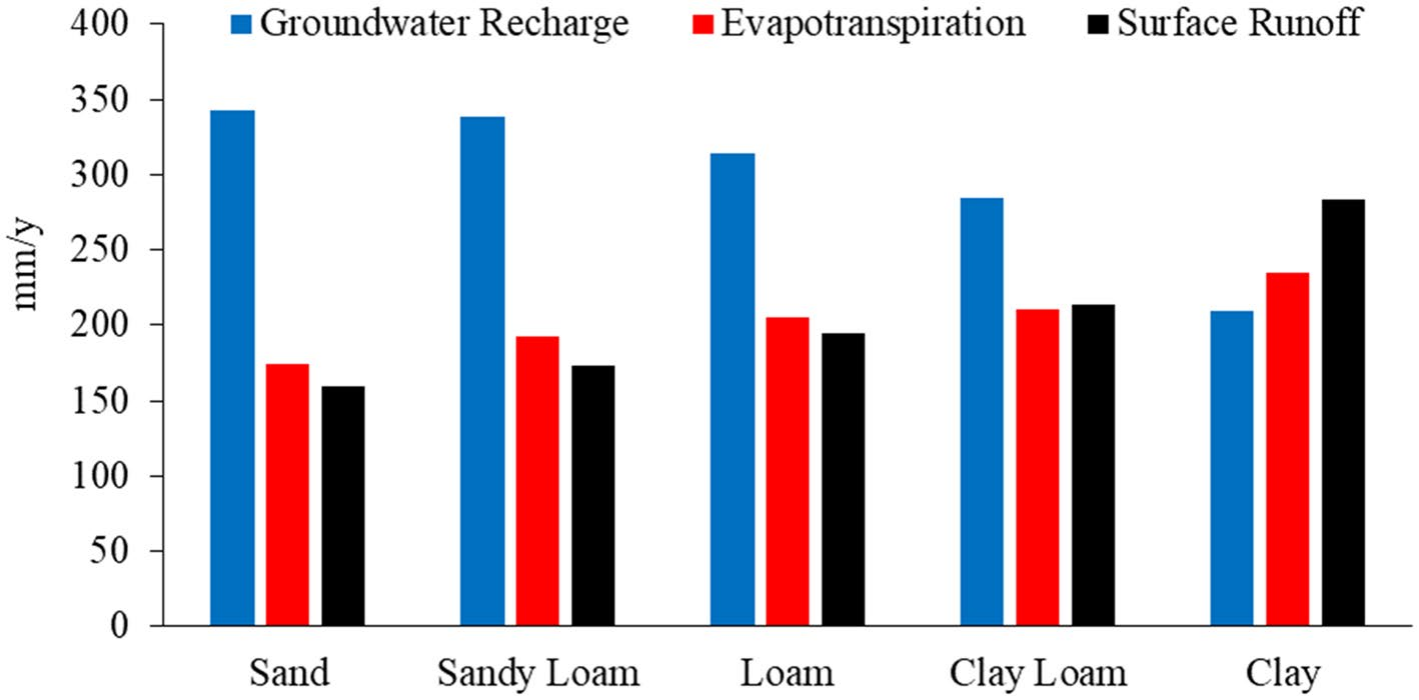
Average annual groundwater recharge, evapotranspiration, and runoff as a function of soil texture for LULC in 2018.

Figure 10 presents the water balance components for 2018 as a function of different soil textures in the Drava floodplain. The spatial variability of soil types comes from fluvial land features and strongly influence local/regional hydraulic properties^4^. Hydraulic conductivity, porosity, specific yield, and capillarity depend on textural properties. For the light soil texture, sandy soils of natural levees show the highest average annual groundwater recharge rate because of their high conductivity, while heavy soils (clay) in the swales between point bar ridges have the lowest rate. The groundwater recharge values for sandy loam, loam, and clay loam fall between the values for sand and clay soils. Evaporation strongly depends on the depth of the groundwater table, which is within 1 m in the study area. Heavy soils (clay and clay loam) in abandoned river channels and the riparian zone have the highest evapotranspiration and surface runoff as they are located in the deepest morphological position with shallow groundwater and intensive capillary rise. In contrast, sand and sandy loam soils have the lowest surface runoff.

Both soil texture and LULC classes influence the water balance components. Table 4 shows the average annual groundwater recharge (mm) across different combinations of land-use and soil types to assess the spatial variations of the groundwater recharge as a function of LULC class and soil textures in the Drava floodplain. Higher groundwater recharge was observed in forest and arable areas with sandy soils due to the high permeability of sandy textured soil. On the contrary, clay and clay loam soils dominant areas have shown a lower groundwater recharge because of the less permeability and shallow groundwater system of such soils. The highest average groundwater recharge was accounted to forest areas with sandy soil type, while mud flat land with clay soils shows the lowest average groundwater recharge followed by built-up areas (Table 4). The results indicated that groundwater recharge was more affected by land-use than soil as the standard deviation of the groundwater recharge for the different land-use classes is higher than the standard deviation of groundwater recharge for the different soil textures (Table 4). Similar findings of groundwater recharge variability with soil textures and LULC classes to the present finding have been reported for the Guishui River Basin, China by Pan et al.^51^, for the San Pedro watershed, USA and Mexico, by Nie et al.^52^, for Flanders (Belgium) by Zomlot et al.^53^ and a catchment in northern Ethiopia by Kahsay et al.^54^ and Gebru and Tesfahunegn^55^. A higher evapotranspiration rate was observed in sites where the predominant cover is a water body, mudflat, grassland, and forest with clay loam and clay soils that could be due to high transpiration demand of vegetation cover and water availability of soil type (Table S4). The highest amount of surface runoff was detected in built-up areas with clay soil texture characterized by lower infiltration capacity (Table S5). In contrast, sand and sandy loam soils with forest, meadow, orchard, and shrub land have the lowest amount of surface runoff because of their high permeability (Table S5). Moreover, the present results indicated that LULC variability affects the spatial variability of surface runoff more than the effect of soil type in the study area (Table S5).

**Table 4:** Average annual groundwater recharge across different combinations of soil texture and LULC in 2018.

LULC classes	Soil texture						
	Sand	Sandy loam	Loam	Clay loam	Clay	Average	St.dev
Built-up	231	208	197	181	149	193	27
Industry	184	177	162	149	126	159	21
Infrastructure	275	–	218	195	157	211	43
Agriculture	344	297	279	245	190	271	52
Meadow	393	340	319	266	206	305	64
Orchard	366	–	306	270	210	288	57
Deciduous forest	302	272	255	240	235	261	24
Coniferous forest	250	–	232	–	–	241	9
Mixed forest	284	261	241	236	230	250	20
Shrub	139	161	167	180	183	166	16
Reference		–	276		180	228	48
Mud flat	143	127	123	116	94	121	16
River	0	0	0	0	0	0	0
Lake	–	–	0	–	–	0	0
Average	243	205	198	189	163		
St.dev	107	97	97	76	63		

–, (no value) as there is no such LULC for a given soil texture.

### Impacts of impervious cover changes on water balance components

The share of built-up areas of Drava floodplain did not change much between 1990 and 2012. From 2012 to 2018, the built-up areas increased from 3.2 to 6.1%, mostly converting from arable land. Thus, change in recharge, evapotranspiration, and surface runoff under built-up areas for this period were assessed to assess the urbanization impacts on the water balance (Table 5). Evapotranspiration and surface runoff both increased while groundwater recharge decreased from 2012 to 2018.

**Table 5:** Annual water balance components in the Drava floodplain under built-up areas from 2012 to 2018.

Parameter	Value	Recharge (mm year^–1^)	Evapotranspiration (mm year^–1^)	Surface runoff (mm year^–1^)
2012	Range	137–358	133–383	158–301
	Average	214	211	249
	Standard deviation	53	22	37
2018	Range	133–218	211–260	232–328
	Average	193	227	263
	Standard deviation	21	13	20
Variation		− 10%	+ 7%	+ 6%

### Effect of LULC changes on groundwater quantity and average groundwater level

**Figure 11:**
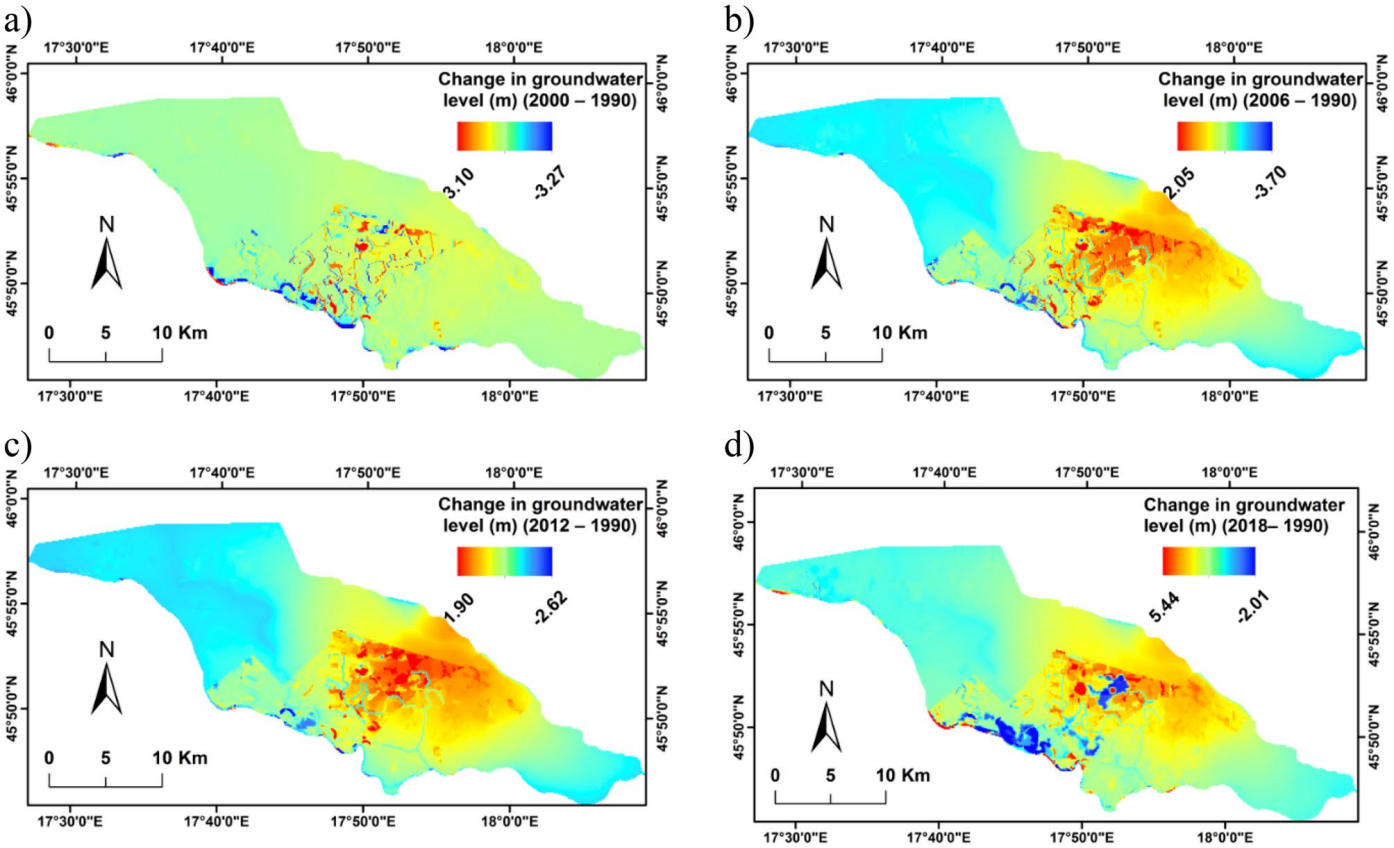
Change in groundwater level of the simulated LULC scenarios with respect to the base case (1990): (**a**) 2000–1990; (**b**) 2006–1990; (**c**) 2012–1990; and (**d**) 2018–1990.

To assess the impacts of the LULC changes on groundwater quantity, a groundwater flow model for each of the years (1990, 2000, 2006, 2012, and 2018) was built using the WetSpass-M results of groundwater recharge and evapotranspiration. The water balance parameters for simulated LULC scenarios are shown in Table 6. Aquifer recharge from precipitation, recharge from the rivers to the aquifer, discharge from the aquifer to the river, evapotranspiration, inflow, and outflow through, and changes in groundwater levels comparing to the base scenario (1990) were used as indicators to quantify the impact of LULC changes on the water budget of the Drava floodplain. The spatial distribution effect of the LULC change (1990–2018) on the groundwater level of the floodplain for all scenarios compared to the base case (1990) is presented in Fig. 11.

**Table 6:** Water balance components (in m^3^ day^−1^) and changes in average groundwater level with respect to the base case (in m).

Scenario	Inflow through GHB boundary	Outflow through GHB boundary	Discharge from aquifer to river	Aquifer recharge from rivers	Aquifer recharge from precipitation	Evapotranspiration	Average change in GW level (m)
1990	812,061	293,247	1,482,103	498,063	550,555	83,287	0.0
2000	812,977	292,507	1,469,941	499,736	536,821	86,139	− 0.04
2006	812,396	293,948	1,488,173	497,428	557,846	83,557	0.02
2012	812,235	294,675	1,494,416	496,679	563,799	81,612	0.06
2018	813,408	294,696	1,472,552	502,269	538,317	84,095	− 0.04

The results clearly reveal a cycle of rising and falling averages of groundwater level for the different scenarios of LULC changes of the floodplain. The variations in groundwater recharge, evapotranspiration and groundwater level were associated with changes in LULC classes. Compared to LULC in 1990, in 2000 recharge rate from precipitation to aquifer decreased by 13,743 m^3^ day^−1^, while actual evapotranspiration increased by 2852 m^3^ day^−1^ (Table 6). Consequently, the mean groundwater level decreased by 0.04 m (Fig. 11a). With respect to the scenario of year 2006, recharge rate from precipitation to aquifer increased by 443,869 m^3^ day^−1^ (Table 6), while average groundwater levels rise of 0.02 m compared to LULC in 1990 (Fig. 11b). Regarding the scenario for 2012, the average groundwater levels increase by 0.06 m compared to the base scenario (Fig. 11c). In the 2018 scenario, the groundwater recharge from precipitation dropped by 25,482 m^3^ day^−1^ and the evapotranspiration increased by 2483 m^3^ day^−1^ with respect to scenario 2012 (Table 6). Thus, the lower amount of groundwater recharge and higher value of evapotranspiration for the current scenario (2018) led to average groundwater levels to be reduced by − 0.1 m over this period. The average groundwater levels decrease by − 0.04 m between 2018 and the base case 1990 (Fig. 11d). Dropping groundwater levels reduced the variety of crops which could be grown in the Drava floodplain and thus reduced the profitability of agriculture. Consequently, to protect the extremely valuable but also vulnerable environment of the Drava floodplain, effective and sustainable management of water resources is required through an accurate assessment of LULC change impact on the hydrological process.

### Sustainable development strategy

The National Framework Strategy on Sustainable Development (NFSSD)^56^ noted the overexploitation of natural resources, the decrease in their quantity and the degradation of their quality. To improve the status, it asks local governments to the foster environmental awareness, to use renewable energy sources, and to implement restricting requirements for resources in a critical state. The proportion of built-up areas compared to the area of the country, the proportion of ecological farming areas in the country, urban environmental quality and the natural capital index are described as indicators. The vegetation-based natural capital index of Hungary is 9.9% which shows that the country has already lost 90% of its natural ecosystem services or uses it for other purposes.

About regional planning: the National Rural Development Strategy (NRDS) set the goals of protecting landscape diversity, biodiversity and the natural resources (soil, water reserves) being crucial for farming, and of maintaining their quantity and quality. The development of regional landscape management plans by involving those affected by land use and the elaboration of a landscape character protection programme (harmonizing general landscape protection with the agricultural strategy by assessing how the landscape protection and nature conservation aspects can be incorporated) are among the strategic directions and actions. The national strategies are in harmony with the goals of European Union agricultural policy. The Drava being a transboundary river, a common strategy of sustainable use of the environment has also been proposed within the framework of the Transboundary Biosphere Reserve Mura-Drava-Danube (TBR MDD). A main goal of the strategy is the coordination of development activities. The Biosphere Reserve provides a strong international framework for developing common protection and management of the area’s unique natural values and forming a catalyst for sustainable development in the region. It will have a common zonation plan (core, buffer and transition zones). It will facilitate the sharing of information, skills and staff between both countries and the development of collaborative protection, management, research and sustainable development and restoration projects.

## Conclusions

In a floodplain environment under aridification, the most important criterion by which the sustainability of land use/land cover and its changes can be judged, is the impact of the individual LULC on water budget, both surface and subsurface. This paper simulates the impacts of LULC changes from 1990 to 2018 on the annual average water balance components and groundwater levels of the Drava floodplain. Five LULC scenarios (1990, 2000, 2006, 2012, and 2018) were considered. The groundwater recharge represents 48% of precipitation, while both evapotranspiration and surface runoff collectively make up 26%. The expansion of artificial (sealed) surfaces between 2012 and 2018 was detected as the main contributor to the increase of surface runoff by 9 mm year^−1^ in the Drava basin. Changes of arable land, forest, mudflat, and willow shrubs were observed as having a negative effect on groundwater recharge and a positive impact on actual evapotranspiration. The replacement of arable and meadow lands by forest, mudflat, with expanding willow shrub areas was identified as the strongest contributor to the major increase in evapotranspiration between LCLU in 1990–2000 and 2012–2018, respectively. These changes in LULC have resulted in a decline of groundwater recharge over an area of 5.3 × 10^7^ m^3^, amounting to 0.1 m drop of groundwater level regarding the whole floodplain between 2012 and 2018. A decrease in groundwater recharge would directly reduce recharge for both deep and shallow aquifers, and it increased the environmental threat in the floodplain.

Consequently, the conditions of agriculture that represent the primary source of subsistence for the local population became critical. The results also show that LULC changes have a direct effect on groundwater recharge and the provision of floodplain ecosystem services. The coupled model proved to be a valuable assessment tool for effective and sustainable implementation of floodplain rehabilitation plans. As a result of human intervention, available water resources in the Drava floodplain are rapidly diminishing, effective integrated watershed management is required to address water availability. Decision-makers should take measures to minimize the negative effects of further LULC change: restricting surface sealing, encouraging the cultivation of crops with reduced water demand on arable land, preservation of grazing land and meadows, and checking their overgrowth with shrubs.

Finally, we are fully aware of the limitations of our method, which mostly derive from two sources. First, the paper presents the consequences of LULC and climate change in isolation and disregards their interactions and the combined effects, often enhanced by other human impact. Secondly, even if a hydrological model is calibrated and verified using realistic statistical error measures, model uncertainties related to input data and model structure are to be addressed by ensemble runs. A future study might expand on these findings by exploring regional variations in sensitivity to particular LULC changes affecting forests, developed land and different types of crops. Despite those limitations, the findings of the integrated modelling are applicable not only to assess the influence of climate change and LULC on regional hydrological processes in the Drava floodplain, but to any region provided the necessary data are available.

## Supplementary Information

Supplementary Information.

## Data Availability

The datasets used and/or analyzed during the current study available from the corresponding author on reasonable request.

## References

[1] *Impact of Human Activity on Groundwater Dynamics: Proceedings of an International Symposium (Symposium S3) Held During the Sixth Scientific Assembly of the International Association of Hydrological Sciences (IAHS) at Maastricht, The Netherlands, from 18 to 27 July 2001* (IAHS, 2001).

[2] Han D, Currell MJ, Cao G, Hall B (2017). Alterations to groundwater recharge due to anthropogenic landscape change. J. Hydrol..

[3] Tang Z, Engel BA, Pijanowski BC, Lim KJ (2005). Forecasting land use change and its environmental impact at a watershed scale. J. Environ. Manag..

[4] Dezső J, Lóczy D, Salem A, Gábor N, Lóczy D (2019). Floodplain connectivity. The Drava River: Environmental Problems and Solutions.

[5] Bonacci O, Oskoruš D, Lóczy D (2019). Human impacts on water regime. The Drava River: Environmental Problems and Solutions.

[6] Pinto, I. S. *et al*. Final knowledge assessment reports of the 3 case studies and lessons learned. In *Deliverable 3.1 of the EU-FP7-Project KNEU, Contract No. 265299*. 10.13140/RG.2.1.1481.4801 (2013).

[7] Lóczy, D. *et al*. Rehabilitation potential of the Drava river floodplain in Hungary. In *Water Resources and Wetlands*, 21–29. 10.13140/2.1.4324.4802 (2014).

[8] Salem A, Dezső J, El-Rawy M, Lóczy D, Ksibi M (2021). Water Management and retention opportunities along the hungarian section of the Drava River. Recent Advances in Environmental Science from the Euro-Mediterranean and Surrounding Regions.

[9] Lóczy D, Lóczy D (2018). Geological and geomorphological setting. The Drava River: Environmental Problems and Solutions.

[10] Wang B, Jin M, Nimmo JR, Yang L, Wang W (2008). Estimating groundwater recharge in Hebei Plain, China under varying land use practices using tritium and bromide tracers. J. Hydrol..

[11] El-Rawy M (2016). Conjunctive use of groundwater and surface water resources with aquifer recharge by treated wastewater: Evaluation of management scenarios in the Zarqa River Basin, Jordan. Environ. Earth Sci..

[12] Manghi F, Mortazavi B, Crother C, Hamdi MR (2009). Estimating regional groundwater recharge using a hydrological budget method. Water Resour. Manag..

[13] Moon S-K, Woo NC, Lee KS (2004). Statistical analysis of hydrographs and water-table fluctuation to estimate groundwater recharge. J. Hydrol..

[14] Batelaan O, Smedt FD, Gehrels H, Peters J, Leibundgut C (2001). WetSpass: A flexible, GIS based, distributed recharge methodology for regional groundwater modelling. Impact of Human Activity on Groundwater Dynamics.

[15] Manfreda S, Fiorentino M, Iacobellis V (2005). DREAM: A distributed model for runoff, evapotranspiration, and antecedent soil moisture simulation. Adv. Geosci..

[16] Carrera-Hernández JJ, Gaskin SJ (2008). Spatio-temporal analysis of potential aquifer recharge: Application to the Basin of Mexico. J. Hydrol..

[17] Singh G, Saraswat D (2016). Development and evaluation of targeted marginal land mapping approach in SWAT model for simulating water quality impacts of selected second generation biofeedstock. Environ. Model. Softw..

[18] Zhang D, Madsen H, Ridler ME, Refsgaard JC, Jensen KH (2015). Impact of uncertainty description on assimilating hydraulic head in the MIKE SHE distributed hydrological model. Adv. Water Resour..

[19] Gumindoga W, Rientjes THM, Haile AT, Dube T (2014). Predicting streamflow for land cover changes in the Upper Gilgel Abay River Basin, Ethiopia: A TOPMODEL based approach. Phys. Chem. Earth A/B/C.

[20] McColl C, Aggett G (2007). Land-use forecasting and hydrologic model integration for improved land-use decision support. J. Environ. Manag..

[21] Abdollahi K (2017). A distributed monthly water balance model: Formulation and application on Black Volta Basin. Environ. Earth Sci..

[22] Nannawo AS, Lohani TK, Eshete AA (2021). Exemplifying the effects using wetspass model depicting the landscape modifications on long-term surface and subsurface hydrological water balance in Bilate Basin, Ethiopia. Adv. Civil Eng..

[23] Warku F, Korme T, Wedajo GK, Nedow D (2022). Impacts of land use/cover change and climate variability on groundwater recharge for upper Gibe watershed, Ethiopia. Sustain. Water Resour. Manag..

[24] Pálfai, I. *Magyarország holtágai (Oxbows in Hungary)*, 231 (2001).

[25] Lóczy D, Dezső J, Czigány S, Prokos H, Tóth G (2017). An environmental assessment of water replenishment to a floodplain lake. J. Environ. Manag..

[26] Lóczy D, Dezső J, Gyenizse P, Czigány S, Tóth G, Lóczy D (2019). Oxbow lakes: Hydromorphology. The Drava River: Environmental Problems and Solutions.

[27] Salem, A., Dezső, J., El-Rawy, M. & Lóczy, D. Statistical analysis of precipitation trend for Drava flood plain region in Hungary. In *GSRD International Conference, 579th International Conferences on Engineering and Natural Science (ICENS)* 43–47 (2019).

[28] Allen, R. G., Pereira, L. S., Raes, D. & Smith, M. *Crop Evapotranspiration—Guidelines for Computing Crop Water Requirements—FAO Irrigation and Drainage Paper 56* (1998).

[29] Pistocchi, A. *Leaf Area Index (MAPPE Model)*. http://data.europa.eu/89h/jrc-mappe-europe-setup-d-18-lai (European Commission, Joint Research Centre (JRC), 2015). Accessed 10 June 2020.

[30] MTA ATK TAKI. http://mta-taki.hu/osztalyok/gis-labor/agrotopo (2013). Accessed 05 June 2020.

[31] Heymann, Y., Steenmans, C., Croissille, G. & Bossard, M. *CORINE Land Cover. Technical Guide* (2014).

[32] Feranec J, Hazeu G, Christensen S, Jaffrain G (2007). Corine land cover change detection in Europe (case studies of the Netherlands and Slovakia). Land Use Policy.

[33] Kallimanis AS, Koutsias N (2013). Geographical patterns of Corine land cover diversity across Europe: The effect of grain size and thematic resolution. Prog. Phys. Geogr. Earth Environ..

[34] Falťan V (2020). Comparison of CORINE land cover data with national statistics and the possibility to record this data on a local scale—Case studies from Slovakia. Remote Sensing.

[35] Büttner G, Manakos I, Braun M (2014). CORINE land cover and land cover change products. Land Use and Land Cover Mapping in Europe.

[36] Cieślak I, Biłozor A, Szuniewicz K (2020). The use of the CORINE land cover (CLC) database for analyzing urban sprawl. Remote Sensing.

[37] Dams J (2013). Mapping impervious surface change from remote sensing for hydrological modeling. J. Hydrol..

[38] Zomlot Z, Verbeiren B, Huysmans M, Batelaan O (2017). Trajectory analysis of land use and land cover maps to improve spatial–temporal patterns, and impact assessment on groundwater recharge. J. Hydrol..

[39] Batelaan O, De Smedt F (2007). GIS-based recharge estimation by coupling surface–subsurface water balances. J. Hydrol..

[40] Niswonger, R. G., Panday, S. & Ibaraki, M. *MODFLOW-NWT, A Newton Formulation for MODFLOW-2005* (2011).

[41] Winston, R. B. ModelMuse-A graphical user interface for MODFLOW-2005 and PHAST: US Geological Survey techniques and methods 6-A29. In *US Geological Survey*. http://pubs.usgs.gov/tm/tm6A29 (2009). Accessed 10 May 2019.

[42] Dezső HA, Czigány S, Tóth G, Lóczy D (2017). Estimating seepage loss during water replenishment to a floodplain oxbow: A case study from the Drava Plain. Geogr. Fisica e Dinamica Quat..

[43] Salem, A., Dezső, J., Lóczy, D., El-Rawy, M. & Slowik, M. *Modeling Surface Water-Groundwater Interaction in an Oxbow of the Drava Floodplain*, Vol. 3, 1832–1822 (2018).

[44] Salem A, Dezső J, El-Rawy M, Lóczy D (2020). Hydrological modeling to assess the efficiency of groundwater replenishment through natural reservoirs in the Hungarian Drava river floodplain. Water.

[45] Doody, T. M., Benyon, G. & Theiveyanathan, T. Quantifying water savings from willow removal in creeks in south-central NSW. In *9th International River Symposium, Brisbane* (2006).

[46] Yin L (2014). Dynamics of willow tree (*Salix matsudana*) water use and its response to environmental factors in the semi-arid Hailiutu River catchment, Northwest China. Environ. Earth Sci..

[47] Doody TM (2011). Potential for water salvage by removal of non-native woody vegetation from dryland river systems: Water salvage from removal of non-native riparian species. Hydrol. Process..

[48] Marttila H, Dudley BD, Graham S, Srinivasan MS (2018). Does transpiration from invasive stream side willows dominate low-flow conditions? An investigation using hydrometric and isotopic methods in a headwater catchment. Ecohydrology.

[49] Singhal BBS, Gupta RP (2010). Applied Hydrogeology of Fractured Rocks.

[50] Čermák J, Prax A (2001). Water balance of a Southern Moravian floodplain forest under natural and modified soil water regimes and its ecological consequences. Ann. For. Sci..

[51] Pan Y, Gong H, Zhou D, Li X, Nakagoshi N (2011). Impact of land use change on groundwater recharge in Guishui River Basin, China. Chin. Geogr. Sci..

[52] Nie W (2011). Assessing impacts of landuse and landcover changes on hydrology for the upper San Pedro watershed. J. Hydrol..

[53] Zomlot Z, Verbeiren B, Huysmans M, Batelaan O (2015). Spatial distribution of groundwater recharge and base flow: Assessment of controlling factors. J. Hydrol. Reg. Stud..

[54] Kahsay GH (2019). Spatial groundwater recharge estimation in Raya basin, Northern Ethiopia: An approach using GIS based water balance model. Sustain. Water Resour. Manag..

[55] Gebru TA, Tesfahunegn GB (2020). GIS based water balance components estimation in northern Ethiopia catchment. Soil Tillage Res..

[56] NFSSD, National Framework Strategy on Sustainable Development of Hungary. *The National Council for Sustainable Development, Hungary*. http://www.nfft.hu/web/ncsd/documents (2013). Accessed 10 July 2022.

