# Supplementary material for: Integrated assessment of the impact of land use changes on groundwater recharge and groundwater level in the Drava floodplain, Hungary
Source: Sci Rep. 2023 Mar 28;13:5061. doi: 10.1038/s41598-022-21259-4 (PMC10050007; doi:10.1038/s41598-022-21259-4)
Supplement: Supplementary file 1 — Supplementary Information. [file 41598_2022_21259_MOESM1_ESM.docx]

Appendix A

Specific yield measurements

A gravimetric method ^76^ was used to measure the specific yield of undisturbed samples for 72 sites in the investigated area (Fig. 3b). First, the samples were dried in an oven at 105 ºC for 24 hours and filled with water until full saturation. Then, the samples were put on a sand bed for 24 hours so that the gravitational water was lost from the soil. In this period, the amount of capillarity lost from the undisturbed samples, and the difference represents the specific yield. The measured values of specific yield for different lithologies are presented in Table S1. The observed daily groundwater levels for 18 observation wells were obtained from the STWMD. Table S2 presents the coordinates and annual change in groundwater level and specific yield for each of the observation wells.

**Table S1.** The measured specific yield for different lithologies

| **Lithology** | **Specific Yield** |
| --- | --- |
| Sand | 0.27 |
| Sandy Loam | 0.22 |
| Loam | 0.20 |
| Clay Loam | 0.13 |
| Clay | 0.07 |

**Table S2** The location, average annual groundwater depth, and average annual change in groundwater

| **Station** | **Longitude and latitude** | **Average annual groundwater depth (m)** | **Average annual change in groundwater (m)** |
| --- | --- | --- | --- |
| Drávafok | 17⁰45′50″E, 45⁰53′50″N | 3 | 1.35 |
| Sellye | 17⁰50′52″E, 45⁰52′52″N | 3.11 | 1.05 |
| Kemse | 17⁰54′48″E, 45⁰49′48″N | 2.2 | 1.18 |
| Vajszló | 17⁰59′16″E, 45⁰51′16″N | 3.25 | 0.77 |
| Kákics | 17⁰51′22″E, 45⁰54′22″N | 1.4 | 1.7 |
| Lakócsa | 17⁰41′32″E, 45⁰53′32″N | 1.63 | 1.45 |
| Dráva-iványi NY | 17⁰49′3″E, 45⁰50′3″N | 1.5 | 1.6 |
| Vejti NY | 17⁰58′10″E, 45⁰49′10″N | 3.15 | 1.8 |
| Potony | 17⁰39′10″E, 45⁰55′10″N | 2.34 | 1.64 |
| Ketujfalu | 17⁰42′46″E, 45⁰57′46″N | 2.6 | 1.1 |
| CUN_2 | 18⁰5′6″E, 45⁰48′6″N | 3.59 | 1 |
| CUN_3 | 18⁰4′41″E, 45⁰48′41″N | 1.66 | 1.18 |
| CUN_5 | 18⁰4′43″E, 45⁰46′43″N | 2.3 | 1.22 |
| CUN_1 | 18⁰4′44″E, 45⁰47′44″N | 1.69 | 1.7 |
| CUN_4 | 18⁰5′46″E, 45⁰47′46″N | 3.19 | 1.8 |
| Szaporca K-8 | 18⁰6′12″E, 45⁰46′12″N | 2.78 | 0.6 |
| Darány | 17⁰35′20″E, 45⁰58′20″N | 1.69 | 1.53 |
| Barcs | 17⁰27′17″E, 45⁰57′17″N | 4.82 | 0.8 |

**Table S3** The statistical analysis (min, max, average, standard deviation) for LULC scenarios of 1990, 2000, 2006, 2012, and 2018

| **Scenario** | **Value** | **Recharge (mm)** | **Runoff (mm)** | **Evapotranspiration (mm)** |
| --- | --- | --- | --- | --- |
| **Land-use 1990** | **Min-Max** | 0-533 | 80-680 | 50-1393 |
|  | **Average** | 325 | 183 | 180 |
|  | **Std. dev.** | 100 | 70 | 140 |
| **Land-use 2000** | **Min-Max** | 0-533 | 42-756 | 79-1435 |
|  | **Average** | 317 | 177 | 200 |
|  | **Std. dev.** | 100 | 80 | 159 |
| **Land-use 2006** | **Min-Max** | 0-533 | 80-680 | 50-1393 |
|  | **Average** | 329 | 182 | 177 |
|  | **Std. dev.** | 100 | 71 | 142 |
| **Land-use 2012** | **Min-Max** | 0-533 | 80-680 | 49-1393 |
|  | **Average** | 333 | 180 | 169 |
|  | **Std. dev.** | 100 | 71 | 138 |
| **Land-use 2018** | **Min-Max** | 0-533 | 80-680 | 50-1393 |
|  | **Average** | 317 | 189 | 192 |
|  | **Std. dev.** | 106 | 80 | 170 |

**Table S4** Average annual actual evapotranspiration across different combinations of soil texture and LULC in 2018

| **LULC classes** | **Soil texture** | | | | | | | |
| --- | --- | --- | --- | --- | --- | --- | --- | --- |
|  | **Sand** | **Sandy Loam** | | **Loam** | **Clay Loam** | **Clay** | **Average** | **St.dev** |
| **Built-up** | 218 | 227 | 237 | | 248 | 258 | 238 | 14 |
| **Industry** | 219 | 221 | 234 | | 242 | 254 | 234 | 13 |
| **Infrastructure** | 217 | - | 241 | | 254 | 267 | 245 | 18 |
| **Agriculture** | 168 | 196 | 204 | | 215 | 220 | 201 | 18 |
| **Meadow** | 165 | 190 | 202 | | 235 | 236 | 206 | 27 |
| **Orchard** | 153 | - | 186 | | 195 | 189 | 181 | 16 |
| **Deciduous forest** | 257 | 311 | 344 | | 346 | 392 | 330 | 45 |
| **Coniferous forest** | 362 | - | 411 | | - | - | 386 | 25 |
| **Mixed forest** | 269 | 331 | 359 | | 371 | 406 | 347 | 46 |
| **Shrub** | 395 | 347 | 335 | | 296 | 239 | 322 | 52 |
| **Reference** | - | - | 190 | | - | 219 | 205 | 15 |
| **Mud flat** | 477 | 511 | 526 | | 543 | 573 | 526 | 32 |
| **River** | 1345 | 1330 | 1356 | | 1357 | 1358 | 1349 | 11 |
| **Lake** | - | - | 1371 | | - |  | 1371 | 0 |
| **Average** | 354 | 407 | 443 | | 391 | 384 |  |  |
| **St.dev** | 314 | 340 | 388 | | 320 | 312 |  |  |

***- (no value) as there is no such LULC for a given soil texture***

**Table S5** Average annual surface runoff across different combinations of soil texture and LULC in 2018

| **LULC classes** | **Soil texture** | | | | | | | |
| --- | --- | --- | --- | --- | --- | --- | --- | --- |
|  | **Sand** | **Sandy Loam** | | **Loam** | **Clay Loam** | **Clay** | **Average** | **St.dev** |
| **Built-up** | 225 | 233 | 252 | | 260 | 297 | 253 | 25 |
| **Industry** | 280 | 271 | 297 | | 307 | 337 | 298 | 23 |
| **Infrastructure** | 180 | - | 218 | | 238 | 279 | 229 | 36 |
| **Agriculture** | 174 | 196 | 209 | | 235 | 293 | 222 | 41 |
| **Meadow** | 118 | 142 | 155 | | 177 | 242 | 167 | 42 |
| **Orchard** | 143 | - | 178 | | 211 | 258 | 198 | 42 |
| **Deciduous forest** | 101 | 120 | 139 | | 167 | 229 | 151 | 45 |
| **Coniferous forest** | 105 | - | 154 | | - | - | 130 | 24 |
| **Mixed forest** | 103 | 132 | 145 | | 175 | 239 | 159 | 46 |
| **Shrub** | 141 | 162 | 177 | | 200 | 259 | 188 | 41 |
| **Reference** | - | - | 213 | | - | 288 | 251 | 38 |
| **Mud flat** | 207 | 218 | 237 | | 255 | 299 | 243 | 32 |
| **River** | 160 | 161 | 162 | | 162 | 162 | 161 | 1 |
| **Lake** | - | - | 162 | | - | - | 162 | 0 |
| **Average** | 161 | 182 | 193 | | 217 | 265 |  |  |
| **St.dev** | 53 | 48 | 45 | | 44 | 43 |  |  |

***-, (no value) as there is no such LULC for a given soil texture***

| **a)**  **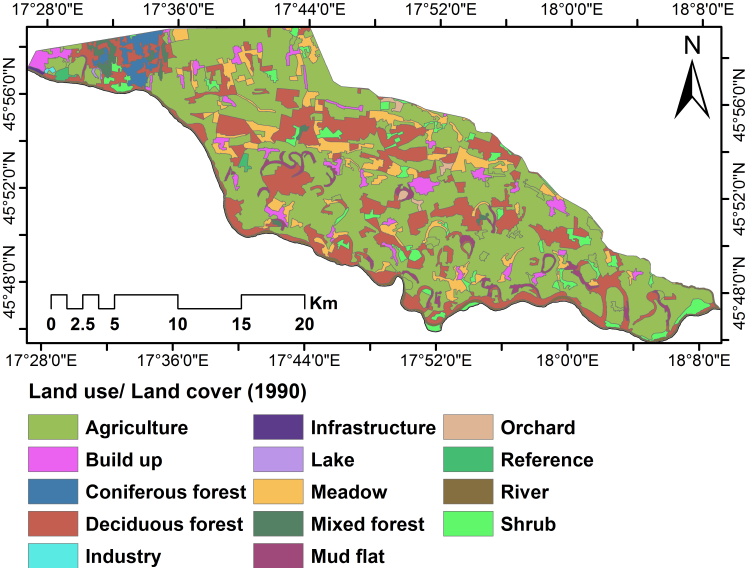** | **b)**  **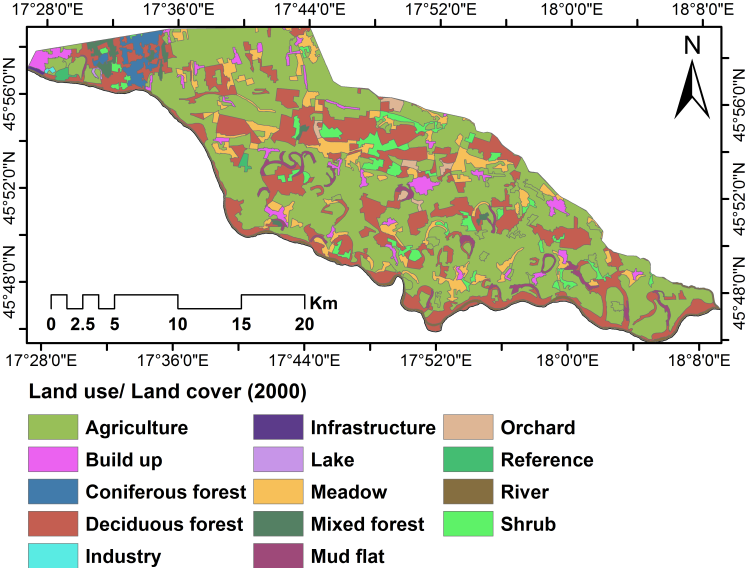** |
| --- | --- |
| **c)**  **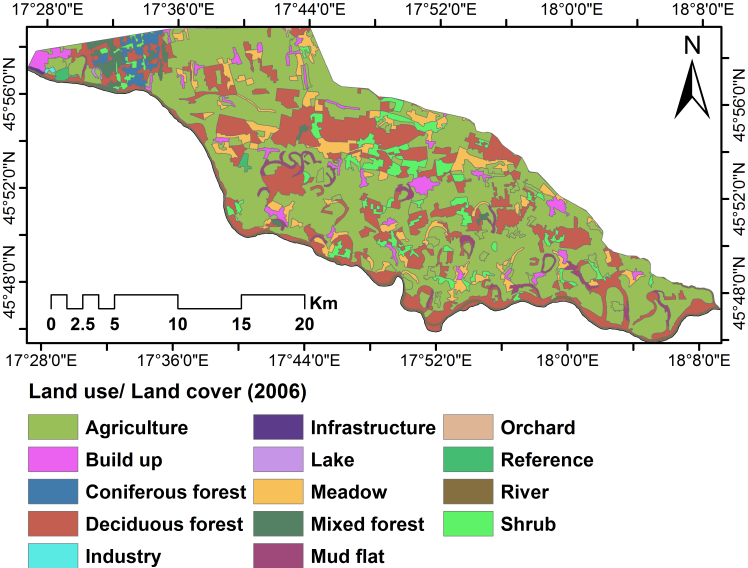** | **d)**  **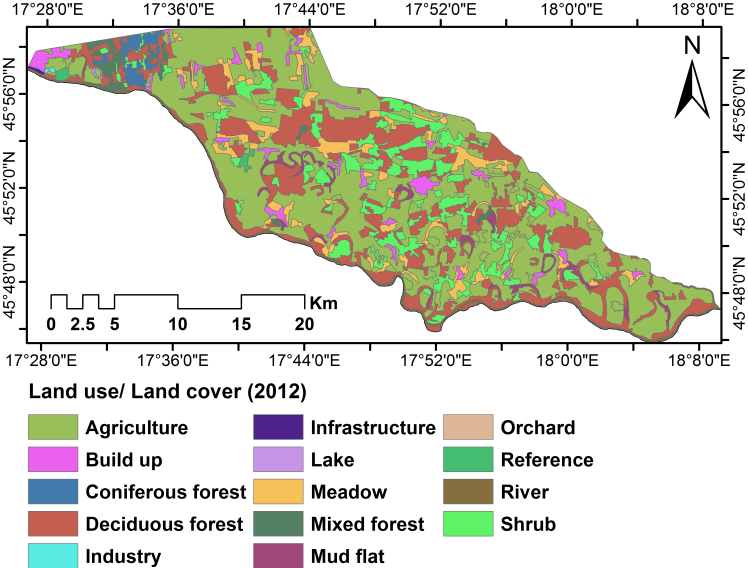** |
| **e)**  **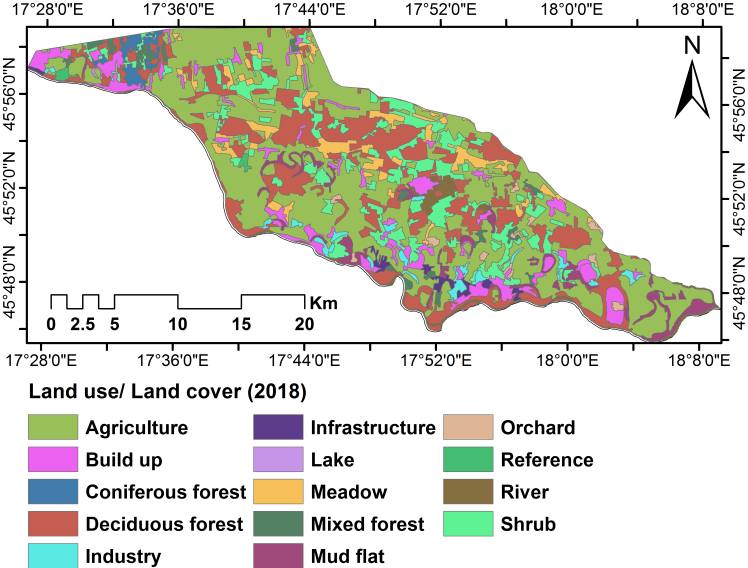** |  |

**Fig. S1**. Land use/ Land cover of the study area at: (a) 1990; (b) 2000; (c) 2006; (d) 2012; and (e) 2018.

| **a)**  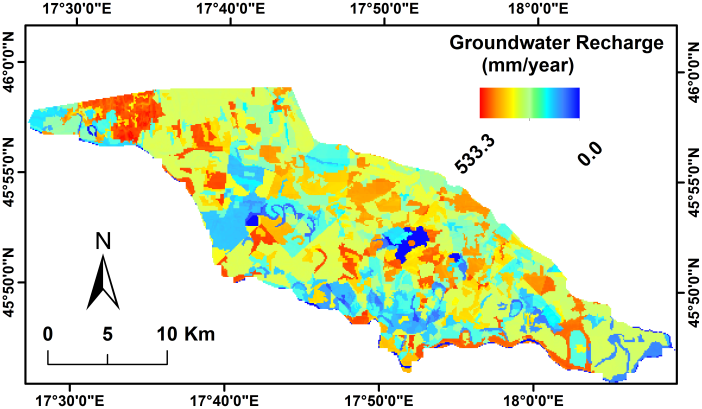 | **b)**  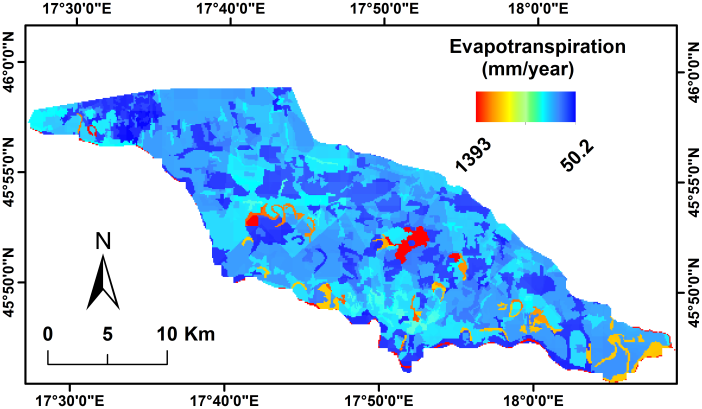 |
| --- | --- |
| **c)**  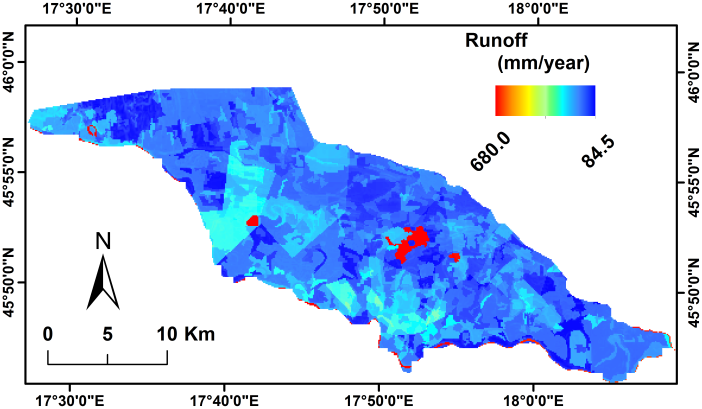 | **d)** 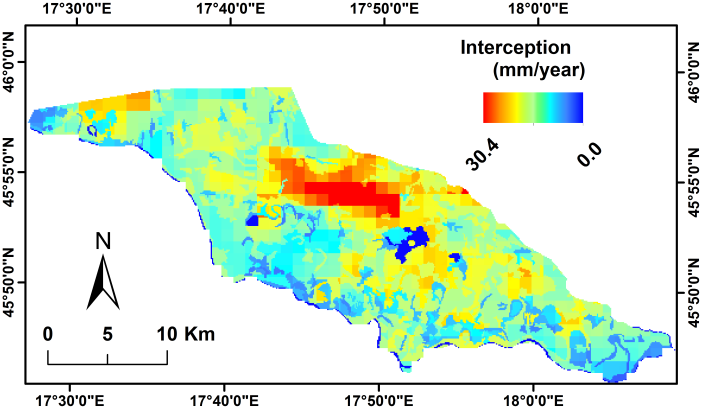 |
| **e)**  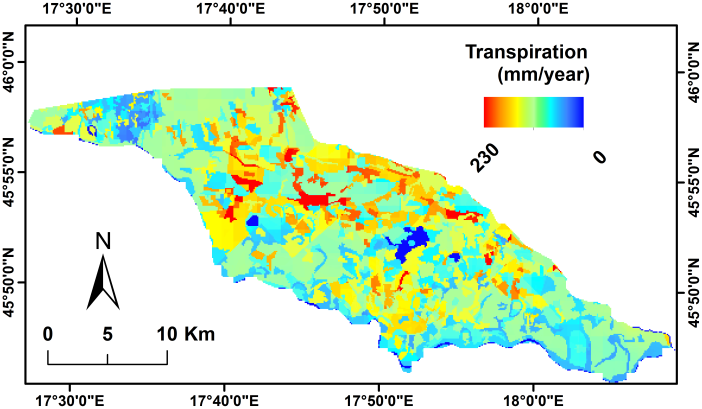 |  |

**Fig. S2**. Spatial distribution of annual average water balance components for LULC in 2018: (a) groundwater recharge; (b) actual evapotranspiration; (c) surface runoff; and (d) interception; and (e) transpiration

| **a)**  **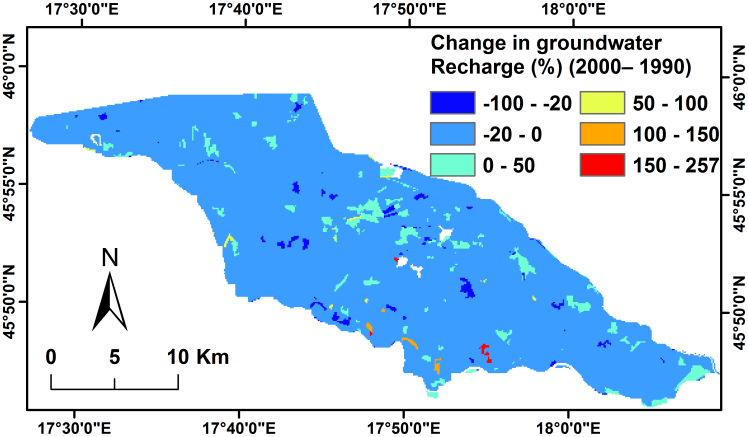** | **b)**  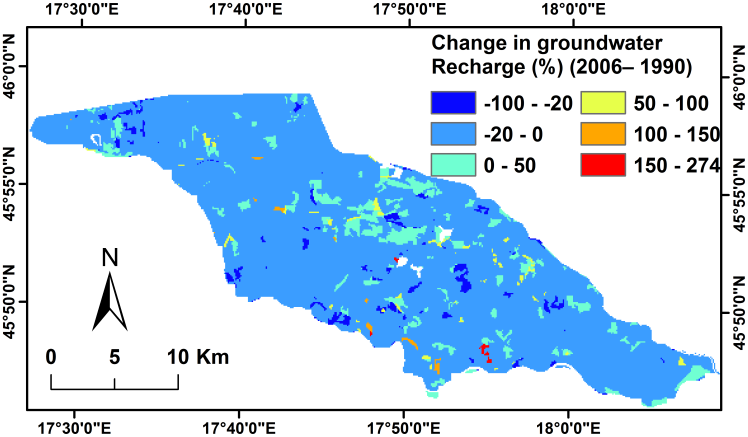 |
| --- | --- |
| **c)**  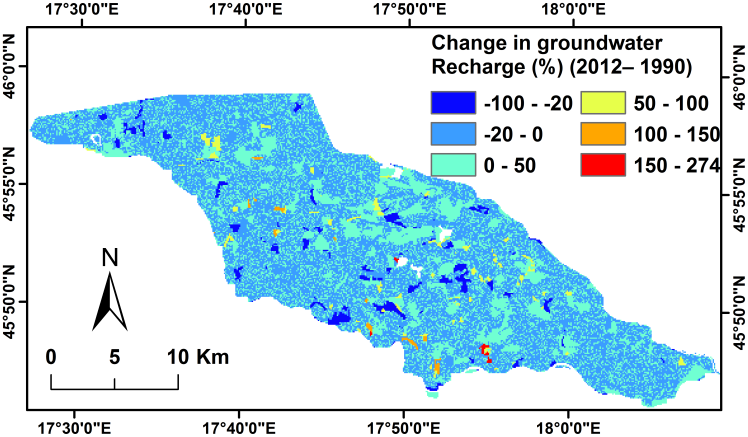 | **d)**  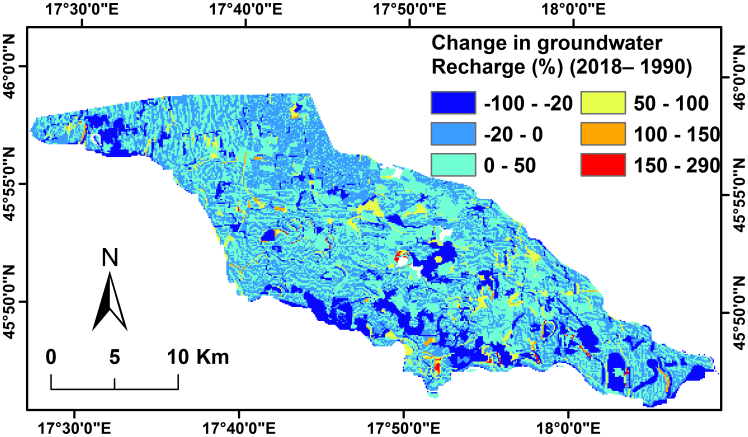 |

**Fig. S3.** Change in groundwater recharge of the simulated LULC scenarios with respect to the base year 1990: (a) 2000 – 1990; (b) 2006 – 1990; (c) 2012 – 1990; and (d) 2018 – 1990
